# Ultra-fast MRI for brain-age prediction in a real-world cognitive disorders clinic

**DOI:** 10.3389/fnagi.2026.1731909

**Published:** 2026-03-18

**Authors:** Rafael Navarro-González, Rodrigo de Luis-García, Santiago Aja-Fernández, Wei Liu, Daniel C. Alexander, Frederik Barkhof, Millie Beament, Haroon R. Chughtai, Nick C. Fox, Catherine J. Mummery, Miguel Rosa-Grilo, David L. Thomas, Geoff J. M. Parker, James H. Cole

**Affiliations:** 1 Laboratorio de Procesado de Imagen, Universidad de Valladolid, Valladolid, Spain; 2 Instituto de Investigación Biosanitaria de Valladolid, IBioVALL, Valladolid, Spain; 3 Research and Clinical Translation, Magnetic Resonance, Siemens Healthineers AG, Erlangen, Germany; 4 Department of Computer Science, Hawkes Institute, UCL, London, United Kingdom; 5 Dementia Research Centre, UCL Queen Square Institute of Neurology, UCL, London, United Kingdom; 6 Radiology and Nuclear Medicine, Amsterdam UMC, Vrije Universiteit, Amsterdam, Netherlands; 7 Centre for Advanced Research Computing, UCL, London, United Kingdom; 8 Department of Translational Neuroscience and Stroke, UCL Queen Square Institute of Neurology, UCL, London, United Kingdom; 9 Bioxydyn Limited, Manchester, United Kingdom

**Keywords:** Alzheimer’s disease, brain-age, dementia diagnosis, disease modifying therapies in Alzheimer’s disease, structural MRI, Wave-CAIPI

## Abstract

**Introduction:**

Alzheimer’s trials and memory-clinic workflows require frequent structural MRI, but standard 3D-T1 MPRAGE can be burdensome and motion-prone. The Wave-CAIPI sequence offers major time savings, yet it is unclear whether these ultra-fast scans can be used to derive dementia-related biomarkers from models that have been trained on standard scans.

**Methods:**

We acquired paired scans from the standard and Wave-CAIPI MPRAGE protocols in 147 patients from a cognitive disorders clinic and generated measures of the brain’s biological age. We applied six public brain-age pipelines (brainageR, DeepBrainNet, PyBrainAge, ENIGMA, pyment, MCCQR-MLP) to assess variability across software packages. We evaluated accuracy, interchangeability, cross-protocol agreement and clinical discrimination (subjective memory complaints versus neurodegenerative disorders), and tested effects of acquisition, diagnosis, and its interaction in a mixed-effects model.

**Results:**

Cross-protocol agreement was excellent across brain-age pipelines (intraclass correlation coefficient: ICC ≳ 0.90). Clinical discrimination was comparable between protocols, with effect sizes varying modestly by model-protocol combinations. Small, model-specific offsets and significant acquisition-by-diagnosis interactions were seen for some pipelines, consistent with a calibratable protocol effect; test–retest reliability was high and quality control measures were similar across protocols.

**Discussion:**

The ultra-fast Wave-CAIPI protocol could generate robust brain-age estimates in memory clinic patients, while markedly reducing scan time. When mixing ultra-fast and standard scans, a harmonization or transfer learning step is advisable to remove model-dependent offsets.

## 1 Introduction

Magnetic resonance imaging (MRI) underpins research and clinical care for neurodegenerative diseases, providing high-resolution anatomical information for diagnosis, characterization, and monitoring (Mugler and Brookeman, 1990; Frisoni et al., 2010; Biondo et al., 2022; Verdi et al., 2023; Høilund-Carlsen et al., 2024). In routine workflows and trials, the widely used 3D T1-weighted MPRAGE sequence offers excellent brain tissue contrast but its relatively long duration (typically above 5 min) can cause discomfort for patients, impacting tolerance of scanning and increasing the risk of motion artifacts, particularly in older adults and those with cognitive impairment (Savalia et al., 2017; Nguyen et al., 2020). In addition, elevated in-scanner head motion has been reported in individuals with mild cognitive impairment and Alzheimer’s disease compared with elderly controls (Haller et al., 2014). Importantly, even subtle head motion during structural MRI acquisition has been shown to bias cortical thickness and volumetric estimates derived from T1-weighted images (Reuter et al., 2015).

Because imaging-derived biomarkers are increasingly used to track disease and treatment effects in Alzheimer’s disease and related disorders (ADRD), scans are often repeated over time for both safety and efficacy monitoring (Baecker et al., 2021; Cummings et al., 2021; Barakos et al., 2022). Recent acceleration strategies, such as Wave-Controlled Aliasing in Parallel Imaging MPRAGE (Wave-CAIPI MPRAGE), could drastically reduce scan times without sacrificing image quality (Bilgic et al., 2015; Polak et al., 2018). Additionally, novel acceleration strategies, including AI-enabled reconstruction algorithms and compressed sensing, have further enhanced both efficiency and quality of MRI scans (Chen et al., 2022; Priyanka et al., 2024).

Early studies have shown that Wave-CAIPI MPRAGE produces volumetric measures and visual ratings comparable to standard MPRAGE sequences, supporting its clinical utility (Goncalves Filho et al., 2020; Longo et al., 2020; Rosa-Grilo et al., 2025). However, going beyond volumetric assessment, there has been growing interest in applying machine-learning models to T1-weighted MRI for the estimation of brain-health biomarkers and the prediction of diagnostic labels or clinical outcomes in ADRD (Pellegrini et al., 2018; Ahmadzadeh et al., 2023; Martin et al., 2023). Brain-age, an MRI-derived estimate of the brain’s biological age, has emerged as a particularly promising biomarker in this context (Franke et al., 2010; Cole and Franke, 2017; Gaser et al., 2024), with the brain-age gap showing sensitivity to neurodegenerative disease and predictive value for cognitive decline and dementia risk (Wang et al., 2019; Biondo et al., 2022; Lee et al., 2022; Wrigglesworth et al., 2022; Wittens et al., 2024; Papouli and Cole, 2025). Despite comparable volumetric measures, Wave-CAIPI MPRAGE can differ from conventional MPRAGE in k-space sampling and noise characteristics due to its use of controlled aliasing and parallel imaging (Bilgic et al., 2015), which may induce a domain shift relative to the data used to train existing brain-age models. These acquisition-dependent differences have been associated with modest reductions in signal-to-noise ratio and subtle changes in image sharpness, which prior work on accelerated T1-weighted protocols has shown can introduce small but systematic biases in automated segmentation and morphometric analyses (Sakurama et al., 2022). This concern is reinforced by evidence that brain-age estimates can be model-dependent, with different algorithms producing different absolute values and downstream associations despite high reliability, for example, showing divergent effect sizes or clinical associations depending on the modeling approach (Bacas et al., 2023). Conversely, if ultra-fast MRI can be shown to be compatible with established brain-age pipelines, this would substantially improve clinical feasibility by enabling near–real-time brain-age estimation with < 2 min of scanning time and reducing motion-related artifacts.

Here, we assessed the use of brain-age in ultra-fast MRI using a paired, within-subject design in a real-world cohort from a cognitive disorders clinic. Each participant underwent both standard MPRAGE and ultra-fast Wave-CAIPI MPRAGE. Since there is no consensus on the optimal brain-age pipeline, we applied six widely used brain-age pipelines spanning voxel-wise and region-based approaches. We aimed to 1) assess the consistency and reliability of brain-age predictions across acquisition protocols, and 2) determine whether ultra-fast and standard acquisitions offer equivalent power to distinguish patients with neurodegenerative disorders from those with milder cognitive concerns across models. We hypothesize that the models will demonstrate comparable performance across acquisition types, supporting the broader clinical adoption of ultra-fast Wave-CAIPI MPRAGE sequences for diagnostic and therapeutic monitoring.

## 2 Materials and methods

### 2.1 Cross-sectional and short-interval datasets

Between November 2022 and February 2024, we recruited 157 individuals who were undergoing brain-MRI as part of their clinical assessment at the Cognitive Disorders Clinics of the National Hospital for Neurology and Neurosurgery, Queen Square, London, United Kingdom. Participants were prospectively enrolled without formal exclusion criteria; however, they needed to be both willing and able to undergo MRI scanning (e.g., free of severe claustrophobia or cognitive impairment that would preclude scanning). Following quality control (described below), 10 participants were excluded, resulting in a final cohort of 147 individuals. This real-world cohort spans a range of cognitive complaints and neurodegenerative diagnoses (mainly Alzheimer’s disease or frontotemporal lobar degeneration, see Rosa-Grilo et al., 2025 for more details). This inclusive recruitment strategy reflects routine memory-clinic populations and allows assessment of brain-age robustness across heterogeneous cognitive presentations.

All participants underwent a standard 3D T1w MPRAGE sequence, following the Alzheimer’s Disease Neuroimaging Initiative 2 (ADNI-2) protocol (Jack et al., 2015), and one or two ultra-fast Wave-CAIPI MPRAGE acquisitions using a research package (acquisition parameters are shown in Table 1). During the study, minor changes were made to the Wave-CAIPI MPRAGE timing parameters. For participants with multiple Wave-CAIPI scans, one acquisition was randomly selected to avoid possible bias from overrepresenting specific subsets of scans. Imaging was performed on a 3T scanner (MAGNETOM Prisma, Siemens Healthineers, Forchheim, Germany) using a 64-channel array head/neck receiver coil. Additional details on the images are provided in Supplementary Table 1.

**TABLE 1:** Demographic characteristics of the included individuals.

Dataset	Clinical group	n	Sex (F/M)	Age (μ ± σ)
Cross-sectional	Subjective memory complaints	72	38/34	57.17 ± 8.93
	Neurodegenerative disorder diagnosis	75	36/39	66.12 ± 8.78
Short-interval	Subjective memory complaints	6	0/6	64.22 ± 6.83
	Neurodegenerative disorder diagnosis	9	2/7	68.21 ± 7.06
	**Parameters**		**Standard MPRAGE**	**Wave-CAIPI MPRAGE**
	Resolution		1.05 × 1.05 × 1.2 mm^3^ (sagittal)	1.1 mm^3^ isotropic (sagittal)
	TR/TI(ms)/flip angle		2,300/900/9°	2,550–2,650/800–900/9°
	Acceleration factor		GRAPPA, R = 2	Wave-CAIPI 3 × 2
	Scan time		5 min 12 s	1 min 34 s– 1 min 37 s

Acquisition parameters for the standard MPRAGE images, following the ADNI-2 protocol, and the ultra-fast Wave-CAIPI MPRAGE images from the cross-sectional dataset (TR, repetition time; TI, inversion time; GRAPPA, GeneRalized Autocalibrating Partially Parallel Acquisitions).

Cognitive function was assessed using the Mini-Mental State Examination (MMSE). Clinical diagnoses incorporated all investigations from the clinical assessment. Participants were divided into two groups: those with subjective memory complaints (SMC), defined as individuals reporting cognitive concerns without clinical diagnoses or imaging evidence of neurodegenerative, vascular, or structural brain abnormalities; and those with neurodegenerative disorders (NDD), defined by clinical diagnoses supported by neuroimaging evidence of underlying pathology. Although simplified, this classification approach remains informative for evaluating general trends and differences between these groups. A second MRI was acquired in 17 participants a mean (SD) of 30.1 (12.2) days after the initial scan. During both sessions, each participant underwent both the standard MPRAGE and the ultra-fast Wave-CAIPI MPRAGE sequences. Two participants were excluded during quality control, leaving 15 for analysis.

### 2.2 Ethical approval and informed consent

Written consent was obtained for all participants and demographic data collected prior to scanning. Ethical approval was granted by the NHS Health Research Authority London (REC reference 21/LO/0815). The study was performed in accordance with the 1964 Declaration of Helsinki and its later amendments.

### 2.3 Brain-age models

This assessment included brain-age software packages that are publicly available, with preprocessing steps, software and model weights accessible online without any restrictions. A related study has previously evaluated the performance of several pipelines examined in this work (Dörfel et al., 2023).

The following packages were evaluated: brainageR (Biondo et al., 2022; Clausen et al., 2022; Hobday et al., 2022), DeepBrainNet (Bashyam et al., 2020), PyBrainAge (Rutherford et al., 2022; Cole, 2025), ENIGMA (Han et al., 2021), pyment (Leonardsen et al., 2022), and MCCQR-MLP. For this last model, as there is no standalone reference available, we provide a full description in Supplementary material. Table 2 outlines a comparison of these packages.

**TABLE 2:** Comparison of brain-age software packages.

Package	Algorithm	Features	Subjects	Age range	Age, μ (σ)	MAE	r	*R* ^2^
brainageR	GPR	Voxel based	3,377	18–92	40.6 (21.4)	4.90	0.947	–
DeepBrainNet	2D CNN	Voxel based (DL)	11,729	3–95	–	4.12	–	–
PyBrainAge	ETR	Region based	29,175	2–100	46.9 (24.4)	4.70	0.660	0.420
ENIGMA(males/females)	RR	Region based	2,188(952/1,236)	18–75	43.32 (15.42)/38.97 (15.68)	6.50/6.84	0.854/0.850	–
Pyment	SFCN	Voxel based (DL)	53,542	3–95	–	3.90	0.975	0.940
MCCQR-MLP	MLP	Region based	4,960	6–94	40.10 (20,42)	5.81	0.930	0.850

For each model we report the training-set characteristics (sample size, age range, mean ± SD age). MAE, Pearson correlation (r), and the coefficient of determination (*R*^2^), are shown for an out-of-sample test of the models if available. GPR, Gaussian process regressor; 2D CNN, 2-dimensional convolutional neural network; ETR, extra-trees regressor; RR, ridge regression; SFCN, simple fully connected network; MLP, multilayer perceptron.

1. brainageR (Biondo et al., 2022): brainageR, leverages Gaussian process regression and employs T1w images processed with SPM12 (Penny et al., 2011) for segmentation into gray matter, white matter, and cerebrospinal fluid (CSF) probability maps. It calculates spatially normalized parameters and applies principal component analysis (PCA) to the combined gray matter, white matter, and CSF probability maps. The first 435 PCA components are utilized for brain-age prediction. The model was trained on 3,377 healthy participants from seven publicly-available datasets acquired in scanners from different vendors, protocols and field strengths, aged 18–92 with an average age of 40.6 years and a standard deviation of 21.4 and is implemented in R.

2. DeepBrainNet (Bashyam et al., 2020): The DeepBrainNet model is a deep learning framework for brain-age prediction using T1w MRI scans. It was trained on a diverse dataset of 11,729 scans spanning ages 3–95 and various geographic locations, scanners, and protocols. The model uses minimal preprocessing, leveraging an inception-resnet-v2 architecture, and achieves high accuracy with a mean absolute error (MAE) of 4.12 years in an out- of-sample cohort.

3. PyBrainAge (Cole, 2025): The PyBrainAge model is a machine-learning tool designed to estimate brain-age from structural T1w MRI scans. It was trained on data from 29,175 healthy individuals (51.1% female), collected across 76 different imaging sites, spanning an extensive age range of 2–100 years with an average age of 46.9 years and a standard deviation of 24.4 years. While younger cohorts were derived from multiple publicly available datasets, older participants predominantly came from subsets of the UK Biobank (*n* = 17,479) (Sudlow et al., 2015). A detailed description of the training is provided in Supplementary material of the work by Rutherford et al. (2022). University of Michigan datasets were excluded. The model utilizes 187 neuroimaging features, including cortical thickness and subcortical volumes, extracted using FreeSurfer’s Destrieux atlas parcellations. An Extra Trees regression algorithm was employed for age prediction, achieving a MAE of 4.7 years and an R-squared value of 0.42. The PyBrainAge tool is implemented in Python and requires the FreeSurfer segmentation output for its predictions (Fischl, 2012).

4. ENIGMA (Han et al., 2021): ENIGMA is a robust multisite brain-age prediction model leveraging structural features derived from FreeSurfer. The algorithm, based on ridge regression, was trained on data from 2,188 healthy controls (952 males and 1,236 females) aged 18–75, spanning 16 and 22 scanners, respectively. The model demonstrated generalizability on an independent out-of-sample dataset of healthy controls, achieving a MAE of 6.50 years for males and 6.84 years for females.

5. Pyment (Leonardsen et al., 2022): pyment is a model based on the Simple Fully Convolutional Network (SFCN) (Peng et al., 2021), predicts brain-age directly as a continuous variable using T1w MRI data. For this work we used the SFCN-reg variant. The model was trained on a large reference dataset (*n* = 53,542; 51.8% female), composed of T1w MRI scans from 21 non-overlapping, publicly available datasets, acquired in different scanners, protocols and field strengths, of healthy participants aged between 3 and 95 years. While younger participants (3–30 years) were derived from multiple datasets, the older age range (45–82 years) predominantly featured scans from the UK Biobank dataset (*n* = 40,330) (Sudlow et al., 2015). Pyment achieved state-of-the-art performance with a MAE of 3.90 years on an external dataset.

6. MCCQR-MLP (see Supplementary material): This Multi-Layer Perceptron model performs quantile regression with Monte Carlo Dropout to assess aleatory and epistemic uncertainty (Hahn et al., 2022). Trained on 4,960 individuals aged 6–94 years from various publicly available datasets, it uses 100 selected features from an initial set of 1,578 extracted via FastSurfer (Henschel et al., 2020). Features are harmonized using ComBat (Johnson et al., 2007), including age, estimated total intracranial volume, and sex as covariates. A full description of the method is in Supplementary Figure 1 and Supplementary Table 2.

### 2.4 Quality control

Quality control for preprocessing and segmentations was conducted using a combination of visual inspection and established tools tailored to each model. Models relying on FreeSurfer or FastSurfer segmentations, including PyBrainAge, ENIGMA, the preprocessing pipeline of pyment, and MCCQR-MLP, underwent quality control using the fsqc tool (Esteban et al., 2017; Deep-Mi/fsqc, 2025) alongside visual inspections. If a scan failed the FreeSurfer or FastSurfer pipeline, it was reprocessed; persistent failures led to exclusion. For brainageR and DeepBrainNet, preprocessing steps were visually inspected to ensure accuracy. After quality control, 10 participants were excluded for poor segmentation or other preprocessing errors: six failures involved only the standard MPRAGE scan, two involved only the Wave-CAIPI scan, and two affected both sequences. Of these ten, two belonged to the short-interval subsample, reducing the number of usable follow-up pairs from 17 to 15.

To rule out scan-quality confounds, we extracted the cortical Euler number, a surface-topology metric used as a proxy for structural image quality (Moqadam et al., 2024), from both FreeSurfer 6.0.0 and FastSurfer v2.2.0 reconstructions, averaging the left- and right-hemisphere values for each scan.

### 2.5 Statistical analysis: cross-sectional dataset

Evaluation metrics included MAE, Pearson’s r, and *R*^2^ to assess prediction accuracy and model fit. We also computed *R*^2^ between MMSE and brain-age gap across models and acquisitions to evaluate its relevance to cognitive performance. Metrics were calculated separately for the SMC cohort, our reference group, and the NDD cohort, and each metric is presented with its 95% bootstrap confidence interval (5,000 resamples). We quantified the agreement between brain-age estimates from Wave-CAIPI and standard MPRAGE, assessed in the full cohort, using an intraclass correlation coefficient for absolute agreement (ICC-AA) (Koo and Li, 2016; Bos et al., 2025), together with Pearson’s r. Pearson’s r captures only the strength of the linear association; ICC-AA goes further by partitioning total variance into true between-subject differences and residual noise and by penalizing any systematic bias, thereby indicating whether the two sequences are truly interchangeable.

For a comprehensive analysis, we fitted a robust linear mixed-effects model (RLMM) to the brain-age gap, with robust estimation to reduce the influence of extreme values. Fixed effects included brain-age model, imaging sequence (standard MPRAGE vs. Wave-CAIPI), disease status (SMC vs. NDD), and their two- and three-way interactions, with age, sex, and intracranial volume (ICV) as covariates. To allow model-specific covariate effects, interactions between model and covariates were included. Repeated measurements were handled by subject-specific random intercepts and random slopes for sequence.


$$\begin{array}{l} Brain-age\;gap_{ijk}\\ =\alpha_{k}+\;{\text{β}}_{1}Seq_{ij}+\;{\text{β}}_{2}Diag_{i}+\;{\text{β}}_{3}\left(Seq_{ij}\times Diag_{i}\right)\\ +{\text{β}}_{4}\left(Model\;\times \;Seq_{ij}\right)+\;{\text{β}}_{5}\left(Model\;\times \;Diag_{i}\right)\\ +\;{\text{β}}_{6}\left(Model\times \;Seq_{ij}\;\times Diag_{i}\right)+\;{\text{β}}_{7}Age_{i}+\;{\text{β}}_{8}Sex_{i}+\;{\text{β}}_{9}ICV_{i}\\ +\;{\text{β}}_{10}\left(Age_{i}\;\times \;Model\right)+\;{\text{β}}_{11}\left(Sex_{i}\;\times \;Model\right)\\ +\;{\text{β}}_{12}\left(ICV_{i}\;\times \;Model\right)+\;{\text{μ}}_{0i}+\;{\text{μ}}_{1i}Seq_{ij\;}+\;{\text{ε}}_{ijk} \end{array}$$

$$\left(\mu_{0\,i},\mu_{1\,i}\right)∼N\,\left(0,\Sigma_{u}\right),\varepsilon_{i\,j\,k}∼N\,\left(0,\sigma^{2}\right)$$

Here, *i* indexes participants, *j* indexes scans, and *k* indexes the brain-age algorithms. *Model* is a categorical factor indexing the six brain-age algorithms. Interactions with *Model* allow sequence, diagnosis, and covariate effects to vary across algorithms, while subject-level random intercepts and random slopes for sequence account for within-participant dependence.

For each subject and session we formed the within-pair difference Δ = *brain-age_*Standard MPRAGE*_ – brain-age_*Wave*–*CAIPI*_* which isolates the sequence bias to (i) estimate the acquisition bias for every algorithm, (ii) test whether disease status (NDD vs. SMC) modifies the bias, and (iii) detect any drift between sessions. The Δ was analyzed with a linear mixed-effects model (LMM) which modeled the protocol bias as:


$$\begin{array}{l} {\text{Δ}}_{ijk}=\;\alpha_{k}+{\text{β}}_{1}Diag_{i}+{\text{β}}_{2}Sess_{ij}+{\text{β}}_{3}(Diag_{i}\times Sess_{ij})\\ \;+{\text{γ}}_{1}Sex_{i}+{\text{γ}}_{2}Age_{ij}+{\text{γ}}_{3}ICV_{ij}+{\text{μ}}_{i}+{\text{ε}}_{ijk}, \end{array}$$

μi∼N⁢(0,σu2),εi⁢j⁢k∼N⁢(0,σ2)

where *i* indexes participants, *j* the two sessions, and *k* the six brain-age algorithms. The term α_*k*_ captures the mean bias (standard—Wave) for each algorithm; β_1_ captures any difference in bias between diagnostic groups, β_2_ captures systematic drift between session 1 and session 2, and β_3_ tests whether that drift depends on diagnosis. *γ_1_*, *γ_2_*, *γ_3_* adjust the bias for sex, centered age and intracranial volume, respectively. μ_*i*_ ∼ *N*(0, σ_*u*_^2^) is a participant-level random intercept that absorbs all between-subject variation, and ε_*ijk*_ ∼ *N*(0, σ^2^) is the residual error. Assumptions were checked with the Shapiro–Wilk and Breusch–Pagan tests, Q–Q and residual vs. fitted plots, for normality and heteroscedasticity, respectively. All analyses were carried out using Python (v 3.10.23) statistical packages statsmodels (v0.14.4) and pingouin (v0.5.4) and also R v4.4.3 with libraries *robustlmm*, *nlme* and *emmeans*.

Model assumptions were assessed using standard residual diagnostics. *Post-hoc* contrasts were obtained using *emmeans* and corrected for multiple comparisons using the Benjamini–Hochberg false-discovery-rate procedure (q < 0.05). Effect sizes (Cohen’s *d*) were computed on the raw brain-age gap, and receiver-operating-characteristic analyses (AUC) were used to assess group discrimination.

### 2.6 Statistical analysis: short-interval dataset

To examine short-term test-retest reliability we analyzed the participants who returned for a second visit after baseline. For every algorithm and acquisition type we recomputed MAE, Pearson’s *r* and *R*^2^ with respect to chronological age. Ninety-five-percent confidence intervals were obtained by 5,000-fold non-parametric bootstrap. Agreement between session 1 and session 2 was assessed both within each acquisition protocol and across protocols with ICC-AA and the coefficient of variation (CV).

## 3 Results

### 3.1 Brain-age models accuracy

**FIGURE 1:**
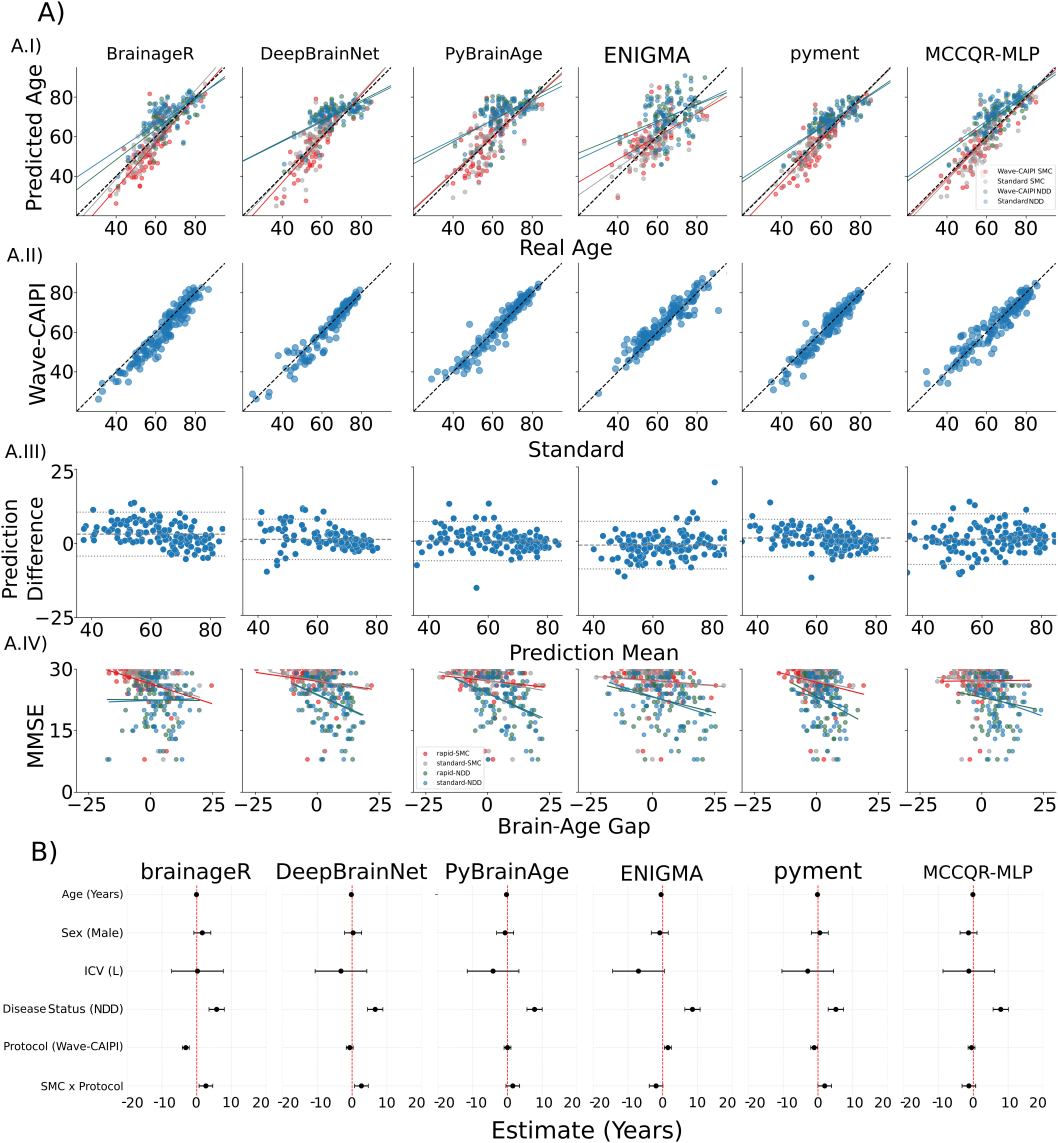
Comprehensive performance summary for the six brain-age models. **(A)** (Column panels) each column corresponds to one model (brainageR, DeepBrainNet, PyBrainAge, ENIGMA, pyment, MCCQR-MLP). **(A.I)** Predicted age versus chronological age; the dashed black line is identity. Points and regression lines are color-coded by acquisition by clinical group: Wave-CAIPI-SMC (red), Wave-CAIPI-NDD (blue), Standard-SMC (gray), Standard-NDD (green). **(A.II)** Direct comparison of Wave-CAIPI against Standard predictions (identity line dashed). **(A.III)** Bland–Altman plots (prediction difference vs. mean); central dashed gray line is average bias, outer gray lines are 95% limits of agreement. **(A.IV)** Brain-age gap versus MMSE, with separate regressions for each colored group. **(B)** (Row panels) forest plots of the linear-model coefficients (predictors: age, sex, intracranial volume, clinical status, acquisition protocol, and their interaction). Black points show estimates with 95% confidence intervals; the red vertical dashed line marks the null effect.

Of the six brain-age prediction models tested on the SMC group, pyment achieved the highest prediction accuracy against chronological age (MAE = 3.73/4.96 years, *r* = 0.88/0.85) while PyBrainAge exhibited the lowest (MAE = 6.69/6.85 years, *r* = 0.69/0.69), for standard/Wave-CAIPI MPRAGE, respectively. For reference, Table 2 lists the out-of-sample performance of each model. Across models, differences in MAE and correlation between standard and Wave-CAIPI acquisitions were generally small; however, two pipelines showed notable protocol-related shifts in the brain-age gap, with Wave-CAIPI yielding lower estimates for brainageR and higher estimates for ENIGMA. These effects are examined in detail in subsequent analyses. All six algorithms showed excellent agreement ICC-AA exceeded 0.90 for every model, and the simple Pearson correlation was likewise > 0.90. Taken together, this indicates that the differences in MAE observed for some models under Wave-CAIPI reflect a systematic shift in predicted age, rather than reduced reliability or loss of rank-order information, as Standard–Wave agreement remained high across pipelines. Euler numbers did not differ between standard and Wave-CAIPI scans (see Supplementary Figure 2). For the standard acquisition, in the SMC group, the brain-age gap had a slight link to MMSE: brainageR accounted for roughly 0.13 of the variance, while every other model explained no more than 0.05 of the variance. In the NDD group PyBrainAge captured 0.16 of the variance, ENIGMA 0.14, while the remaining models stayed below 0.10. These results and values for the Wave-CAIPI acquisition are presented in Figure 1A and Table 3. Results for the NDD group are shown in Supplementary Table 3.

**TABLE 3:** Performance metrics of the evaluated brain-age prediction models for standard MPRAGE and Wave-CAIPI MPRAGE acquisitions in the cross-sectional SMC reference group.

Model	BrainageR	DeepBrainNet	PyBrainAge	ENIGMA	Pyment	MCCQR-MLP
Standard MPRAGE						
MAE	4.92 [4.11, 5.79]	5.27 [4.32, 6.32]	6.69 [5.56, 7.95]	5.85 [4.71, 7.16]	3.73 [3.12, 4.38]	5.98 [5.02, 6.97]
r	0.86 [0.78, 0.91]	0.81 [0.74, 0.88]	0.69 [0.56, 0.79]	0.64 [0.48, 0.78]	0.88 [0.83, 0.93]	0.79 [0.69, 0.87]
*R* ^2^	0.53 [0.14, 0.73]	0.41 [-0.04, 0.64]	0.08 [-0.58, 0.42]	0.21 [-0.38, 0.53]	0.73 [0.52, 0.84]	0.32 [-0.20, 0.59]
MMSE-*R*^2^ SMC	0.11 [-0.08, 0.24]	0.04 [-0.12, 0.11]	0.05 [-0.13, 0.12]	0.01 [-0.10, 0.04]	0.02 [-0.19, 0.07]	0.01 [-0.11, 0.05]
MMSE-*R*^2^ NDD	0.00 [-0.08, 0.01]	0.10 [-0.04, 0.21]	0.16 [-0.03, 0.30]	0.14 [-0.05, 0.25]	0.04 [-0.08, 0.11]	0.06 [-0.06, 0.13]
Wave-CAIPI MPRAGE						
MAE	6.49 [5.45, 7.61]	5.69 [4.69, 6.80]	6.85 [5.68, 8.04]	6.69 [5.46, 7.96]	4.96 [4.10, 5.86]	5.28 [4.35, 6.29]
r	0.82 [0.72, 0.90]	0.81 [0.73, 0.88]	0.69 [0.57, 0.79]	0.56 [0.39, 0.69]	0.85 [0.76, 0.91]	0.80 [0.72, 0.87]
*R* ^2^	0.18 [-0.50, 0.51]	0.33 [-0.25, 0.61]	0.07 [-0.64, 0.42]	0.06 [-0.59, 0.40]	0.50 [0.10, 0.71]	0.43 [-0.03, 0.66]
MMSE-*R*^2^ SMC	0.13 [-0.12, 0.29]	0.03 [-0.11, 0.09]	0.02 [-0.10, 0.06]	0.01 [-0.10, 0.05]	0.05 [-0.11, 0.13]	0.00 [-0.14, 0.00]
MMSE-*R*^2^ NDD	0.00 [-0.08, 0.00]	0.11 [-0.04, 0.22]	0.15 [-0.05, 0.29]	0.08 [-0.07, 0.18]	0.09 [-0.04, 0.19]	0.02 [-0.07, 0.08]
Protocol agreement						
ICC-AA	0.93 [0.72, 0.97]	0.96 [0.94, 0.97]	0.96 [0.95, 0.97]	0.93 [0.89, 0.95]	0.95 [0.93, 0.97]	0.94 [0.91, 0.96]
r Standard vs. Wave-CAIPI	0.96 [0.95, 0.97]	0.96 [0.95, 0.98]	0.96 [0.95, 0.98]	0.94 [0.91, 0.96]	0.96 [0.95, 0.98]	0.94 [0.92, 0.96]

Metrics include MAE, Pearson’s correlation, and *R*^2^ to assess the relationship between predicted and actual ages. Additionally, MMSE-*R*^2^ quantifies the variance explained when regressing the MMSE scores with the brain-age gap. Values are shown as point estimate ± 95% confidence interval; all confidence intervals were obtained by non-parametric bootstrapping with 5,000 resamples. ICC-AA coefficient together with Pearson’s r values, were used to evaluate the agreement between predictions from standard and Wave-CAIPI MPRAGE acquisitions on the whole cross-sectional dataset.

### 3.2 RLMM covariate effects on brain-age gap

Model diagnostics confirmed that the RLMM successfully cushioned the analysis against non-normal data. Although residuals showed statistically detectable departure from normality (Shapiro–Wilk *W* = 0.983, *p* < 0.001), no evidence of heteroscedasticity was observed (non-significant Breusch–Pagan LM = 2.35, *p* = 0.13). Supplementary Figure 3 displays the Q-Q plot and residual-versus-fitted values plot for the RLMM. Robustness weights for residuals and random effects were predominantly near unity across models, with the non-unity weights showing a median of approximately 0.8, indicating that the models effectively down-weighted a small proportion of observations with mild deviations while maintaining overall stability (see Supplementary Table 4). All fixed effects coefficients of the RLMM model are shown in Supplementary Table 5.

Using covariate-adjusted contrasts from the *emmeans post-hoc* analysis of the RLMM, we identified several predictors of the brain-age gap (Figure 1B). Disease status was significantly associated with larger brain-age gaps in the NDD group across all models. Switching to the ultra-fast Wave-CAIPI sequence shifted the mean brain-age gap downward in four of the six models, with the largest and statistically significant effect observed for brainageR (-3.1 years, *q* < 0.01); DeepBrainNet, MCCQR-MLP, and pyment showed non-significant negative trends ( ≤ -1.1 years), while PyBrainAge showed no detectable effect. In contrast, ENIGMA exhibited a significant positive acquisition effect, with Wave-CAIPI increasing the brain-age gap by 1.6 years (*q* < 0.01). Disease-by-acquisition interactions were significant for brainageR and DeepBrainNet (β≈ 2.6–2.7 years, *q* = 0.03), indicating that the disease-related brain-age gap was larger under Wave-CAIPI than under standard MPRAGE for these models; a similar interaction was present at trend level for pyment (β = 2.0 years, *p* = 0.05), whereas no significant interaction effects were detected for PyBrainAge, ENIGMA, or MCCQR-MLP. Age showed a negative association with brain-age gap across all models and reached statistical significance for DeepBrainNet, PyBrainAge, ENIGMA, and pyment, while no statistically significant main effects or interactions involving sex or intracranial volume were observed. These results are summarized in Figure 1B and Table 4. Because the Disease status—protocol interaction was significant for brainageR and DeepBrainNet, we followed up with simple-effects contrasts for each pipeline. In both models the disease status effect remained significant under each acquisition protocol, but it was approximately 2.5 years larger with the Wave-CAIPI sequence than with the standard MPRAGE. Numerical details are provided in Supplementary Table 6.

**TABLE 4:** Regression estimates and *p*-values for the six brain-age models evaluated, brainageR, DeepBrainNet, pyment, PyBrainAge, ENIGMA, and MCCQR-MLP, within the RLMM, across key predictors: Age, Sex, ICV, Disease Status, Acquisition type, and SMC-by-Acquisition Interaction.

Model/metric		Age (years)	Sex (male)	ICV (L)	Disease status (SMC/NDD)	Acq (Wave-CAIPI—Standard)	Disease status × acquisition
BrainageR	Estimate [95 % CI]	–0.09 [–0.20, 0.02]	1.55 [–0.88, 3.99]	0.26 [–7.16, 7.68]	5.65 [3.46, 7.85]	–3.12 [–4.11, –2.14]	2.60 [0.62, 4.57]
	*p*/*q*	0.12/0.12	0.21/0.81	0.94/0.94	**<0.01/<0.01**	**<0.01/<0.01**	**0.01/0.03**
DeepBrainNet	Estimate [95% CI]	–0.21 [–0.32, –0.10]	0.26 [–2.18, 2.70]	–3.19 [–10.61, 4.23]	6.63 [4.43, 8.82]	–0.70 [–1.69, 0.29]	2.70 [0.72, 4.67]
	***p*/*q***	**<0.01/<0.01**	0.83/0.83	0.40/0.67	**<0.01/<0.01**	0.16/0.25	**<0.01/0.03**
PyBrainAge	Estimate [95% CI]	–0.25 [–0.36, –0.14]	–0.59 [–3.03, 1.84]	–4.32 [–11.73, 3.10]	7.79 [5.59, 9.98]	0.04 [–0.95, 1.03]	1.53 [–0.45, 3.51]
	***p*/*q***	**<0.01/<0.01**	0.63/0.81	0.25/0.67	**<0.01/<0.01**	0.94/0.94	0.13/0.16
ENIGMA	Estimate [95% CI]	–0.45 [–0.56, –0.34]	–0.81 [–3.25, 1.63]	–7.15 [–14.57, 0.27]	8.49 [6.30, 10.69]	1.55 [0.56, 2.54]	–1.91 [–3.89, 0.07]
	***p*/*q***	**<0.01/<0.01**	0.52/0.81	0.06/0.35	**<0.01/<0.01**	**<0.01/<0.01**	0.06/0.09
Pyment	Estimate [95 % CI]	–0.15 [–0.26, –0.03]	0.53 [–1.92, 2.96]	–2.86 [–10.28, 4.55]	5.15 [2.95, 7.34]	–1.05 [–2.04, –0.06]	1.96 [–0.02, 3.93]
	***p*/*q***	**0.01/0.02**	0.67/0.81	0.45/0.67	**<0.01/<0.01**	0.04/0.07	0.06/0.09
MCCQR-MLP	Estimate [95 % CI]	–0.11 [–0.22, 0.00]	–1.27 [–3.71, 1.17]	–1.81 [–9.23, 5.60]	7.82 [5.62, 10.01]	–0.55 [–1.54, 0.44]	–1.19 [–3.17, 0.78]
	***p/q***	0.06/0.07	0.31/0.81	0.63/0.76	**<0.01/<0.01**	0.27/0.33	0.24/0.24

Significant predictors are highlighted in bold, indicating robust associations across models.

**FIGURE 2:**
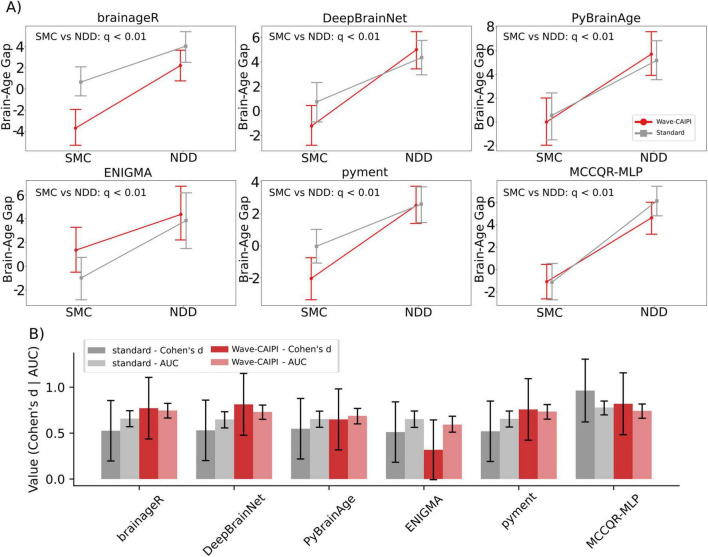
Discrimination between participants with subjective memory complaints (SMC) and neurodegenerative disorders (NDD) across six brain-age pipelines. **(A)** Mean brain-age gap for the SMC and NDD groups. Red lines correspond to Wave-CAIPI MPRAGE acquisitions; gray lines to Standard MPRAGE. Points are group means; error bars show the standard error of the mean (*N* = 147). Statistical annotations indicate the significance of the SMC vs. NDD group comparison for both Standard and Wave-CAIPI acquisitions, based on covariate-adjusted RLMM contrasts. **(B)** Group-separation metrics derived from the same data. For each model the darker bar gives Cohen’s *d* (effect size) and the lighter bar gives the area under the ROC curve (AUC). Red bars: Wave-CAIPI; gray bars: Standard.

Effect-size comparisons between the SMC and NDD groups revealed only modest, model-dependent differences across acquisition protocols. Figure 2 summarizes the raw brain-age gaps for each group under both protocols (Figure 2) and shows the corresponding Cohen’s d and AUC for every model–protocol pairing (Figure 2); the full numerical results are provided in Supplementary Table 7. Supplementary Figure 4 shows the associated ROC curves. Despite these pipeline-specific patterns, the 95% bootstrap confidence intervals for Cohen’s d and AUC overlapped in every comparison, indicating that no acquisition protocol conferred a uniform advantage across all models.

### 3.3 Test-retest reliability and protocol bias

Brain-age predictions showed high test–retest reliability across both acquisition protocols in the short-interval dataset (summarized in Table 5). For Wave-CAIPI in the short-interval dataset, ICC-AA values ranged from 0.94 [95% CI 0.84, 0.98] (pyment) to 0.98 [0.94, 0.99] (brainageR, DeepBrainNet). For Standard MPRAGE, ICC-AA values ranged from 0.92 [95% CI 0.73, 0.97] (MCCQR-MLP) to 0.98 [95% CI 0.94, 0.99] (DeepBrainNet). Within-subject CVs were consistently low across models, ranging from 0.64% (DeepBrainNet, Standard) to 3.29% (ENIGMA, Wave-CAIPI), reflecting stable predictions across repeated scans.

**TABLE 5:** Reliability metrics for brain-age predictions.

Metric/protocol	BrainageR	DeepBrainNet	PyBrainAge	ENIGMA	Pyment	MCCQR-MLP
ICC-AA Wave-CAIPI	0.98 [0.94, 0.99]	0.98 [0.94, 0.99]	0.98 [0.93, 0.99]	0.95 [0.86, 0.98]	0.94 [0.84, 0.98]	0.96 [0.88, 0.99]
CV % Wave-CAIPI	1.54	1.07	1.64	3.29	2.00	1.72
ICC-AA standard	0.94 [0.83, 0.98]	0.98 [0.94,0.99]	0.97 [0.84, 0.99]	0.95 [0.86, 0.98]	0.93 [0.81, 0.98]	0.92 [0.73, 0.97]
CV % Standard	1.87	0.64	1.56	2.85	1.51	2.91
ICC-AA wave-CAIP ↔ standard	0.88 [0.67, 0.96]	0.85 [0.62, 0.95]	0.96 [0.87, 0.98]	0.98 [0.87, 0.99]	0.93 [0.77, 0.98]	0.94 [0.27, 0.99]
CV % wave-CAIP ↔ standard	3.61	2.21	2.15	2.08	1.70	2.68

Intraclass correlation coefficients (ICC-AA) with 95 % confidence intervals and within-subject coefficients of variation (CV %) are shown for each model. Repeatability is provided separately for Wave-CAIPI scans, Standard MPRAGE scans, and for the cross-protocol comparison of the temporal averages of Wave-CAIPI versus Standard scans. ICC-AA denotes absolute agreement.

Cross-protocol agreement between Wave-CAIPI and Standard acquisitions was also high, with ICC-AA values ranging from 0.85 [95% CI 0.62, 0.95] (DeepBrainNet) to 0.98 [95% CI 0.87, 0.99] (ENIGMA). Within-subject cross-protocol CVs were low across models, from 1.70% (pyment) to 3.61% (brainageR).

**FIGURE 3:**
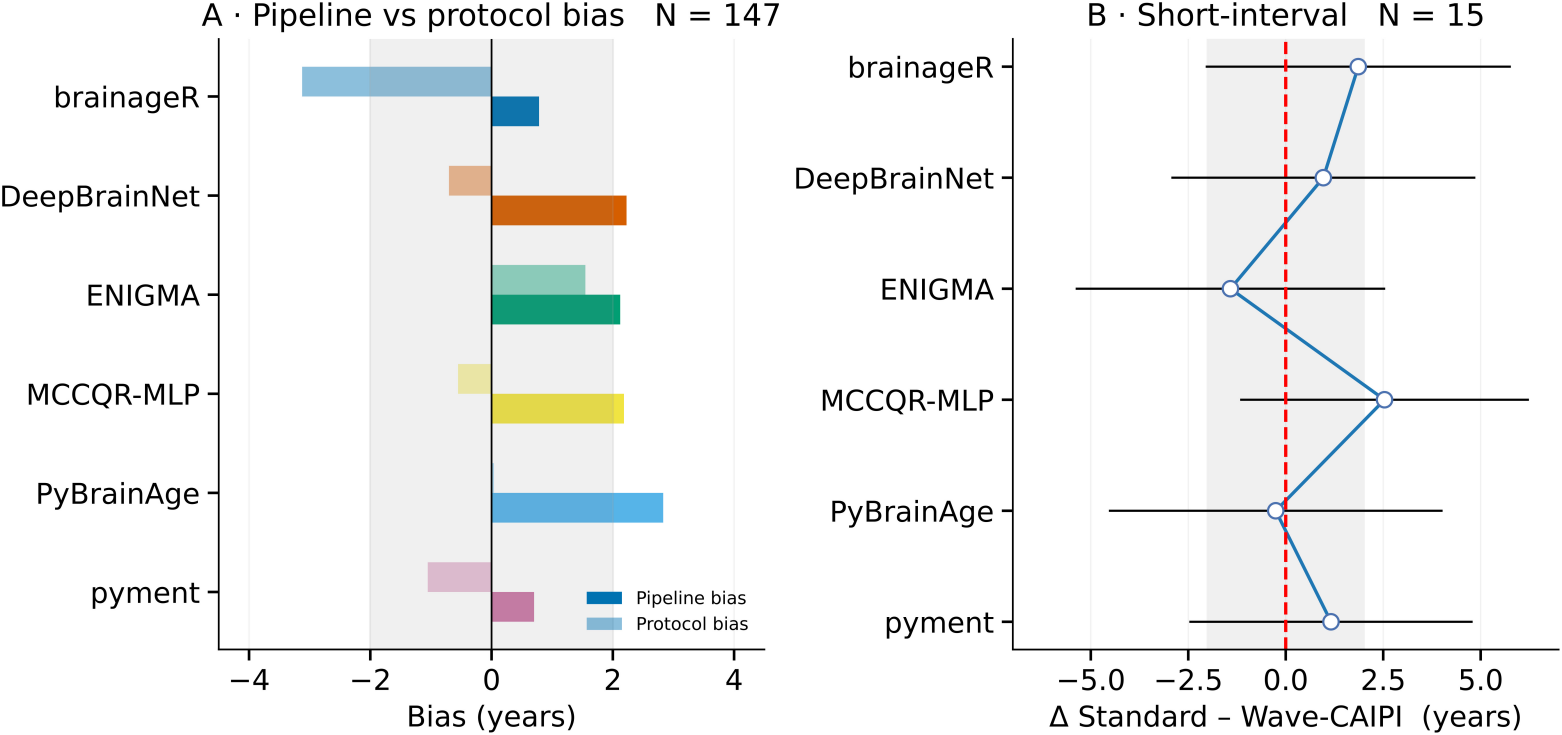
Cross-sectional and longitudinal acquisition bias. **(A)** Cross-sectional cohort (*N* = 147). For each brain-age model the algorithm-specific bias (dark bar) and the protocol bias (light bar) are plotted. Except for brainageR, protocol bias remains within a ± 2 years band and is always smaller than the corresponding pipeline bias. **(B)** Short-interval cohort (*N* = 15). For each participant and session, the within-subject difference was defined as Δ = brain-age_Standard_—brain-age_Wave–CAIPI_. Points show the LMM estimate of the mean within-subject difference (random subject intercept) and lines its 95 % CI, pooling information across both sessions. Including both timepoints increases precision and enables an explicit test for session drift; all CIs span zero and lie within ± 2 years, indicating no detectable within-subject protocol bias.

In the cross-sectional cohort (*N* = 147), we quantified two complementary sources of bias in the RLMM. The Model offset is each pipeline’s covariate-adjusted mean brain-age gap, averaged over both protocols, and the protocol shift is the extra gap that appears when the same pipeline runs on Wave-CAIPI instead of Standard MPRAGE. Related model offsets ranged from 0.70 years (pyment) to 2.83 years (PyBrainAge), while protocol-related shifts ranged from -3.12 years (brainageR) to 1.55 years (ENIGMA). Protocol-related bias was generally smaller than model-specific bias across pipelines, with two exceptions: brainageR, where the acquisition effect (-3.12 years) clearly exceeded the model offset (0.78 years), and pyment, where protocol bias (-1.05 years) was slightly larger than the model bias (0.70 years). However, in pyment, both effects were relatively small. This is shown in Table 4, Supplementary Table 5, and Figure 3A.

In the short-interval longitudinal cohort (*N* = 15), estimated within-subject differences between Standard and Wave-CAIPI acquisitions, via the separate LMM, were not significant. The fixed effects coefficients of the fitted LMM are shown in Supplementary Table 8. Residuals were approximately normal (Shapiro–Wilk W = 1.00, *p* = 0.97), although the Breusch–Pagan test indicated some heteroscedasticity (*p* = 0.03). This was addressed using cluster-robust standard errors. Q-Q plot and residuals vs. fitted plot for the model are shown in Supplementary Figure 3. Point estimates ranged from -1.42 years (ENIGMA) to 2.53 years (MCCQR-MLP), as seen in Figure 3B. Furthermore, performance results (MAE, r, and *R*^2^) for the different brain-age models on the short-interval dataset are shown in Supplementary Table 9. Again, pyment consistently outperformed other models across both protocols, whereas ENIGMA demonstrated comparatively lower accuracy. Protocol-related agreement is shown in the Bland–Altman plots (Supplementary Figure 5), which display within-subject differences between Standard and Wave-CAIPI acquisitions across sessions for each model. BrainageR and pyment exhibited smaller within-subject discrepancies, while ENIGMA and PyBrainAge showed greater variability and protocol-related shifts.

## 4 Discussion

In this study, we examined the influence of Wave-CAIPI MPRAGE versus standard MPRAGE 3D T1w MRI acquisitions from a clinical dataset on the brain-age estimations of six accessible online brain-age models (brainageR, DeepBrainNet, PyBrainAge, ENIGMA, pyment and MCCQR-MLP) and explored how these differences related to clinical status. Among the evaluated models, pyment exhibited the highest predictive accuracy for age (MAE = 3.73 years, *r* = 0.88 for standard MPRAGE; MAE = 4.96 years, *r* = 0.85 for Wave-CAIPI MPRAGE), while PyBrainAge showed the lowest accuracy by MAE and ENIGMA the weakest correlations. For reference, Table 2 contains the out-of-sample results achieved by the models. Across models, agreement between Wave-CAIPI and standard acquisitions was high (ICC-AA typically 0.85–0.98), and all pipelines detected a larger brain-age gap in patients with NDD than in those with SMC, with disease-related differences on the order of several years. Protocol-related shifts were model-dependent but modest, ranging from approximately -3.1 years (brainageR) to +1.6 years (ENIGMA).

Wave-CAIPI scans were just as sensitive as standard MPRAGE for detecting disease effects, with every pipeline still showing a significantly larger brain-age gap in NDD than in SMC, but the magnitude of that gap depended on both protocol and model. In the voxel-wise algorithms (brainageR, DeepBrainNet, pyment) the NDD gap grew by ∼2 years when images were acquired with Wave-CAIPI, yielding a significant acquisition-by-disease interaction for brainageR (β = 2.6 years, *q* = 0.03) and DeepBrainNet (β = 2.7 years, *q* = 0.03), and a trend-level effect for pyment (β = 2.0 years, *q* = 0.09). Region-based methods were more stable: MCCQR-MLP and PyBrainAge showed only small, non-significant interactions, whereas ENIGMA exhibited a borderline acquisition-by-disease interaction (β = -1.9 years, *q* = 0.09) plus a significant main protocol effect (*q* = 0.006), with standard MPRAGE producing a systematically lower brain-age gap by 1.6 years.

In terms of discriminative performance, as measured by AUC and Cohen’s *d*, the protocols perform comparably. A slight advantage is observed for Wave−CAIPI MPRAGE acquisitions in the brainageR, DeepBrainNet, pyment, and PyBrainAge models. Conversely, the ENIGMA and MCCQR−MLP models perform better with the standard MPRAGE protocol. Nevertheless, none of these contrasts are statistically significant: the 95 % confidence intervals for both Cohen’s d and AUC overlap across protocols. Furthermore, deep learning approaches trained on large datasets often achieve the best results in the literature, but they do not appear to outperform other methods in distinguishing between SMC and NDD individuals. Pyment is suggested to have an improved generalization capability (Leonardsen et al., 2022); nevertheless, our findings show that its acquisition fixed effect is still close to significance (*q* = 0.07). Crucially, however, the magnitude of that effect was modest. By contrast, MCCQR-MLP, which integrates a ComBat harmonization layer, showed a negligible protocol term (β = –0.55 years; *q* = 0.33) underscoring how explicit harmonization can reduce acquisition-related drift when datasets are pooled. These observations are consistent with recent ENIGMA work demonstrating that ComBat-GAM harmonization sharpens normative lifespan curves and unveils genotype effects that remain hidden without it (Zhu et al., 2025). In the absence of healthy controls, harmonization parameters were estimated from the SMC group. As a result, some residual disease-related variability may remain in the reference data, and effect sizes may be partially attenuated compared with harmonization derived from a pathology-free cohort.

Taken together, the large-sample analyses indicate a small, model-dependent protocol offset with Wave-CAIPI. In the short-interval test–retest (*N* = 15), within-protocol repeatability was excellent (ICC-AA 0.92–0.98; CV ≤ 3.3%) and cross-protocol agreement likewise high (ICC-AA 0.85–0.98; CV 1.7–3.6%). Consistent with this, the LMM pooling both sessions estimated within-subject Standard–Wave differences whose 95% CIs all spanned zero and were bounded within ± 3 years (Figure 3B). From a clinical perspective, these findings suggest that ultra-fast Wave-CAIPI MPRAGE could be prospectively incorporated into Alzheimer’s disease trial protocols and memory-clinic workflows for repeated structural imaging, provided that simple model-specific harmonization or calibration is applied when mixing acquisition types. Because the shift is modest relative to each model’s 4–8-year MAE and stable across sessions, it behaves like a small, fixed bias amenable to a simple model-specific correction, though this conclusion remains provisional given the modest test–retest sample.

Sex does not appear to influence brain-age predictions significantly. However, age is negatively correlated with the brain-age gap for DeepBrainNet, PyBrainAge, ENIGMA and pyment, indicating that older participants tend to have a smaller brain-age gap than younger ones. This relationship may reflect the “brain-age bias,” where models systematically underestimate brain-age in older individuals and overestimate it in younger ones. Discrepancies among the brain-age models highlight how sample characteristics, scanning protocols, or training age distributions can influence whether a model shows such bias. Addressing brain-age bias via debiasing strategies or incorporating chronological age as a covariate in *post-hoc* analyses is recommended in the literature (de Lange and Cole, 2020).

This study identifies small but significant protocol-related variability in some models, suggesting that acquisition protocols are not fully interchangeable without the application of harmonization or transfer learning techniques. This is unlikely to be an issue relating only to the presence or absence of acquisition acceleration techniques as in this study, but is likely to be observed whenever systematic differences in acquisition protocols exist, such as differences in contrast or resolution. A future study could systematically assess whether different acquisition protocols can be used interchangeably and under what conditions this might be feasible. We did not implement transfer learning, and the deep-learning–based models showed no acquisition-protocol bias in the data; nevertheless, transfer learning remains an intriguing avenue for improving cross-site generalization and robustness in other settings (Wood et al., 2024). We employed ComBat for feature harmonization within the MCCQR-MLP model, which demonstrated good domain adaptation.

Besides Wave-CAIPI, other acceleration strategies, such as compressed sensing and AI-based reconstruction, can also substantially shorten scan time, but they operate through different mechanisms and may introduce distinct forms of bias. Wave-CAIPI accelerates acquisition through controlled aliasing and parallel imaging, primarily altering k-space sampling and noise characteristics, whereas compressed sensing and AI-based approaches rely on sparsity constraints or learned reconstruction priors that can modify image texture, contrast, and intensity distributions. These reconstruction-dependent effects may differentially impact downstream quantitative analyses and machine-learning models. Because we did not compare these acceleration strategies head-to-head within the same dataset, their relative advantages and limitations remain uncertain. Future work should therefore systematically evaluate how different acceleration approaches trade off acquisition speed, image fidelity, and compatibility with existing machine-learning pipelines. Furthermore, a complete assessment of whether ultra-fast Wave-CAIPI and standard MPRAGE scans can be used interchangeably will require multi-site data, as factors such as vendor, field strength, gradient hardware, and software version may interact with both the acquisition protocol and the brain-age model.

One limitation of the present analysis is the difficulty of determining why some brain-age pipelines appear robust to Wave-CAIPI acquisitions while others exhibit protocol-related bias. This sensitivity likely reflects a combination of factors, including differences in preprocessing, feature representations, training data, and implicit scanner adaptation within each model. Wave-CAIPI introduces subtle changes in spatial resolution and image contrast relative to conventional MPRAGE, which may differentially affect pipelines trained on standard acquisitions, particularly region-based methods. Disentangling the relative contributions of these factors will require controlled experiments explicitly varying acquisition and reconstruction parameters; until then, the precise mechanisms underlying model-specific bias remain uncertain.

## 5 Conclusion

In conclusion, this study demonstrates that ultra-fast Wave-CAIPI MPRAGE acquisitions are a viable and effective alternative to standard MPRAGE protocols for brain-age prediction. Across six evaluated models, Wave-CAIPI MPRAGE consistently produced accurate predictions and demonstrated sensitivity to disease-related differences, including significant brain-age gap distinctions between individuals with SMC and NDD. While small differences in model performance were observed, particularly in the interaction effects and protocol-related biases, the overall consistency between protocols highlights the robustness of these models.

The results emphasize the potential of ultra-fast imaging techniques, such as Wave-CAIPI MPRAGE, to reduce scan times without compromising diagnostic accuracy or the capacity to detect disease-relevant changes. Importantly, the substantial reduction in acquisition time achieved with ultra-fast Wave-CAIPI MPRAGE translates into lower patient burden and reduced susceptibility to in-scanner head motion, representing a key practical advantage for memory-clinic workflows and longitudinal Alzheimer’s disease studies. This innovation addresses key limitations of conventional MRI and improves the real-world feasibility of existing brain-age pipelines. However, the variability in model performance underlines the necessity for harmonization techniques or transfer learning to ensure consistency across diverse imaging protocols.

## Data Availability

The datasets for this article are not publicly available because they contain human imaging data that may be identifiable and are subject to ethical and legal restrictions. Requests to access the datasets should be directed to the corresponding author. The code used in this study is available at: https://github.com/rafaloz/BRAPIDD_BA.
