# Supplementary material for: Ultra-fast MRI for brain-age prediction in a real-world cognitive disorders clinic
Source: Front Aging Neurosci. 2026 Mar 18;18:1731909. doi: 10.3389/fnagi.2026.1731909 (PMC13038902; doi:10.3389/fnagi.2026.1731909)
Supplement: Supplementary file 1 [file Data_Sheet_1.docx]

Supplementary Material

|  | **Standard MPRAGE** | **Wave-CAIPI MPRAGE-94** | **Wave-CAIPI MPRAGE-800** | **Wave-CAIPI MPRAGE-800 (WSF)** |
| --- | --- | --- | --- | --- |
| **TI** | 900 ms | 900 ms | 800 ms | 800 ms |
| **TR** | 2300 ms | 2550 ms | 2550 ms | 2650 ms |
| **Acc. factor** | 2 (PE1xPE2 = 2x1) | 6 (PE1xPE2=3x2) | 6 (PE1xPE2=3x2) | 6 (PE1xPE2=3x2) |
| **Resolution** | 1.1x1.1x1.2 mm^3^ | 1.1 mm^3^ isotropic | 1.1 mm^3^ isotropic | 1.1 mm^3^ isotropic |
| **Scanning time** | 312 s | 94 s | 94 s | 97 s |
| **n** | 147 | 46 | 49 | 52 |

**Supplementary Table 1.** TI, inversion time; TR, repetition time; acc. factor, parallel imaging acceleration factor (PE1×PE2 = 3×2, overall factor 6); n indicates the number of subjects. “Standard MPRAGE” refers to the ADNI protocol; MPRAGE‐94, MPRAGE‐800, and MPRAGE‐800 (WSF) are Wave-CAIPI accelerated.

**Monte Carlo Composite Quantile Regression - Multi-Layer Perceptron (MCCQR-MLP)**

**Training Dataset**

To create and evaluate our brain-age prediction model, we compiled a comprehensive dataset consisting of structural T1w MRI scans from a variety of publicly available studies and databases containing only healthy individuals. These include: the Dallas Lifespan Brain Study (DLBS) dataset (Park et al., 2025), the Consortium for Reliability and Reproducibility (CoRR) dataset (Zuo et al., 2014), the Neurocognitive Aging Data Release (NeuroCog) (Spreng et al., 2022), the OASIS-1 Dataset (Marcus et al., 2007), the Southwest University Adult Lifespan Dataset (SALD) (Wei et al., 2018), the Information eXtraction from Images (IXI) dataset (IXI Dataset – Brain Development, n.d.), the CamCAN repository (available at <http://www.mrc-cbu.cam.ac.uk/datasets/camcan/>) (Shafto et al., 2014; Taylor et al., 2017), the National Institute of Mental Health Research Volunteer (NIMH-RV) dataset (Nugent et al., 2022), the Movement-related artifacts (MR-ART) dataset (Nárai et al., 2022), the Nathan Kline Institute - Rockland Sample (NKI-RS) dataset (Nooner et al., 2012), a private dataset from Laboratorio de Procesado de Imagen - UVA. Additionally, we included the AgeRisk dataset (Tisdall et al., 2024) for out-of-sample validation of the model. Supplementary Table 2 provides additional details regarding the dataset.

| **Dataset** | **n** | **Age (years, mean)** | **Age σ** | **Age range** | **Sex (F/M)** | **Scanner B0** |
| --- | --- | --- | --- | --- | --- | --- |
| *CamCan* | 651 | 54.25 | 18.57 | 18-88 | 330/321 | 3.0 T |
| *CoRR* | 1306 | 26.32 | 15.97 | 6-88 | 670/636 | 3.0 T |
| *DLBS* | 315 | 54.62 | 20.09 | 21-89 | 198/117 | 3.0 T |
| *IXI* | 551 | 48.19 | 16.32 | 20-86 | 307/244 | 1.5 T & 3.0 T |
| *LPI* | 258 | 31.47 | 14.31 | 9-86 | 151/107 | 3.0 T |
| *MR-ART* | 148 | 30.01 | 12.76 | 18-74 | 95/53 | 3.0 T |
| *NIMH-RV* | 151 | 34.21 | 12.85 | 18-72 | 99/52 | 3.0 T |
| *NKI-RS* | 479 | 40.60 | 15.62 | 19-83 | 302/177 | 3.0 T |
| *NeuroCog* | 297 | 40.43 | 22.80 | 18-83 | 166/131 | 3.0 T |
| *OASIS-1* | 312 | 44.98 | 23.76 | 18-94 | 196/116 | 1.5 T |
| *SALD* | 492 | 45.16 | 17.47 | 19-80 | 307/185 | 3.0 T |
| *AgeRisk* | 187 | 45.14 | 19.27 | 16-81 | 97/90 | 3.0 T |
| ***Training***  ***Dataset*** | **4960** | **40.10** | **20.42** | **6-94** | **2821/2139** | **1.5T & 3.0T** |

**Supplementary Table 2.** Datasets used for model training and evaluation. All datasets are available online, except for the LPI dataset, available upon request. The row whole dataset sums up all the data used for training. The AgeRisk dataset was used to evaluate the out of sample performance of the model. Datasets have been acquired from different scanners and vendors, with different B0 and acquisition parameters.

**Feature Extraction**

From the T1w images, FastSurfer 2.2.0 (Henschel et al., 2020) was employed to extract a total of 1578 brain-related intensity and morphological features. FastSurfer uses deep learning to perform brain segmentation based on the Desikan-Killiany-Tourville atlas (Alexander et al., 2019). Feature extraction follows a similar scheme to that used in the work by Navarro et al.(Navarro-González et al., 2023). The extracted features were harmonized using ComBat (Johnson et al., 2007) including, age, sex and estimated intracranial volume as covariates.

**Training Procedure**

The training dataset was randomly split into an 8:1:1 ratio for training, validation, and an internal test set for evaluating the machine learning model. Outliers for each feature, defined as values above or below the 97.5th or 2.5th percentile, were flattened. Additionally, all features were scaled to the range (-1, 1) using min-max normalization. Feature selection was performed in three steps to reduce the set to 100 characteristics. First, a filter was applied to select features in the top 20% based on mutual information with age in the training set. Secondly, features with a correlation higher than 0.8 were selected to be removed, retaining only the feature with the best MAE performance based on a random forest regressor. Finally, a forward feature selection method using Gaussian mixture models was applied to further optimize the mutual information between a final subset of features and age (Maia Polo and Vicente, 2023). This procedure was developed using the training set and then applied to the validation and the internal test sets. Lastly, an MLP was trained to perform quantile regression on the features selected from the training split and evaluated on the subsequent splits (parameters: 1 hidden layer 16 neurons, epochs=500, lr=0.01, weight decay=0.01, validation size=0.2, criterion=L1, optimizer=Adam, early stopping=20 epochs). The MLP integrated the uncertainty quantification and calibration procedures presented in (Hahn et al., 2022; Ernsting et al., 2023). The median predicted value is taken as the predicted age for each individual. The training process was carried out using the scikit-learn Python library for machine learning, while the MLP model was implemented using PyTorch.

**Harmonization procedure**

We applied a harmonization approach that involved first estimating harmonization parameters from a reference population, specifically, individuals with SMC, and subsequently applying these learned parameters to a separate group of individuals with NDD. This approach was adopted to reduce scanner-induced variability while minimizing the risk of attenuating biological variability associated with neurodegenerative pathology that is more prevalent in the NDD group. In the absence of healthy controls, the SMC group served as a pragmatic reference representing a comparatively milder clinical subgroup. Although SMC participants did not show overt structural abnormalities on visual inspection, they cannot be assumed to be entirely free of subclinical pathology.

**Results**

The model demonstrates a MAE of 5.11 years, a Pearson r of 0.95, and an R² of 0.89 in the held-out test set. Similarly, in the Out-of-Sample test, applying the model on the AgeRisk dataset, the results show a MAE of 5.81 years, an R² of 0.85, and a Pearson correlation coefficient of 0.93. These performance metrics are shown in Supplementary Figure 1.


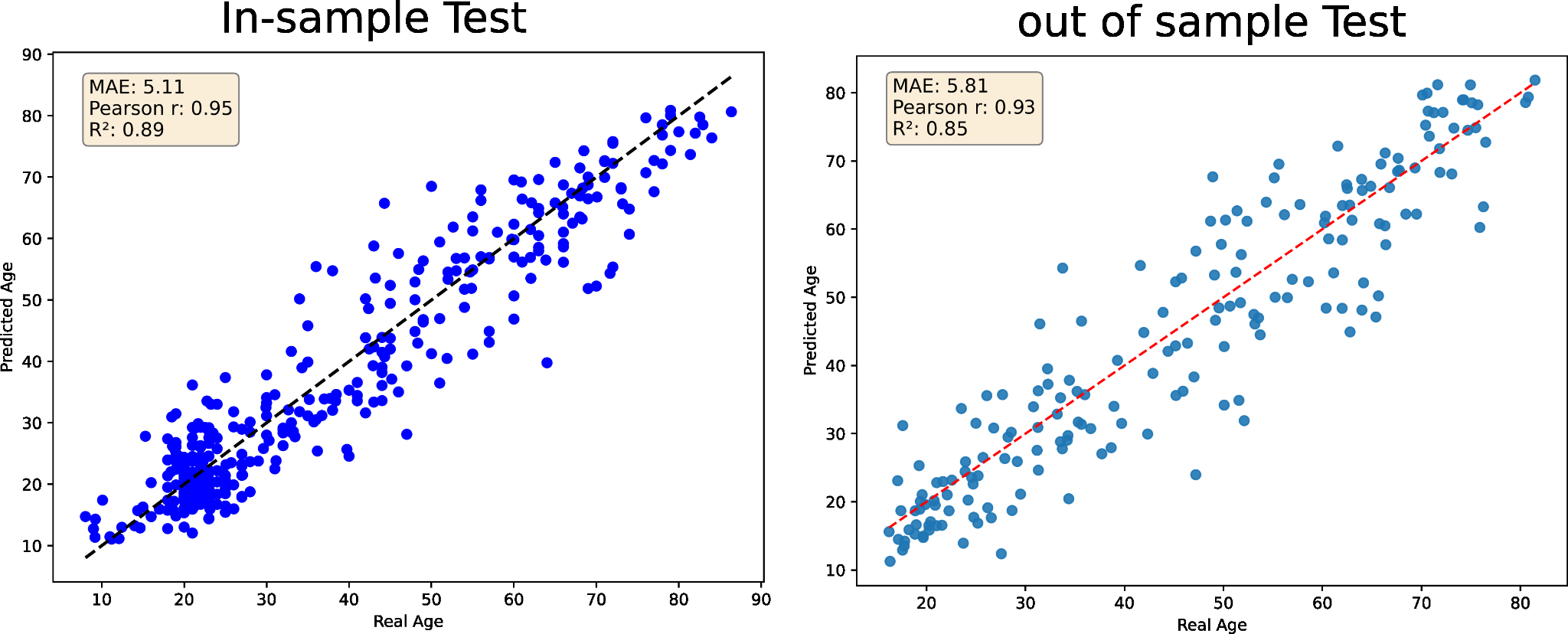


**Supplementary Figure 1.** Scatter plots of predicted vs. real age for the MCCQR-MLP model. Dashed lines represent the line of equality.


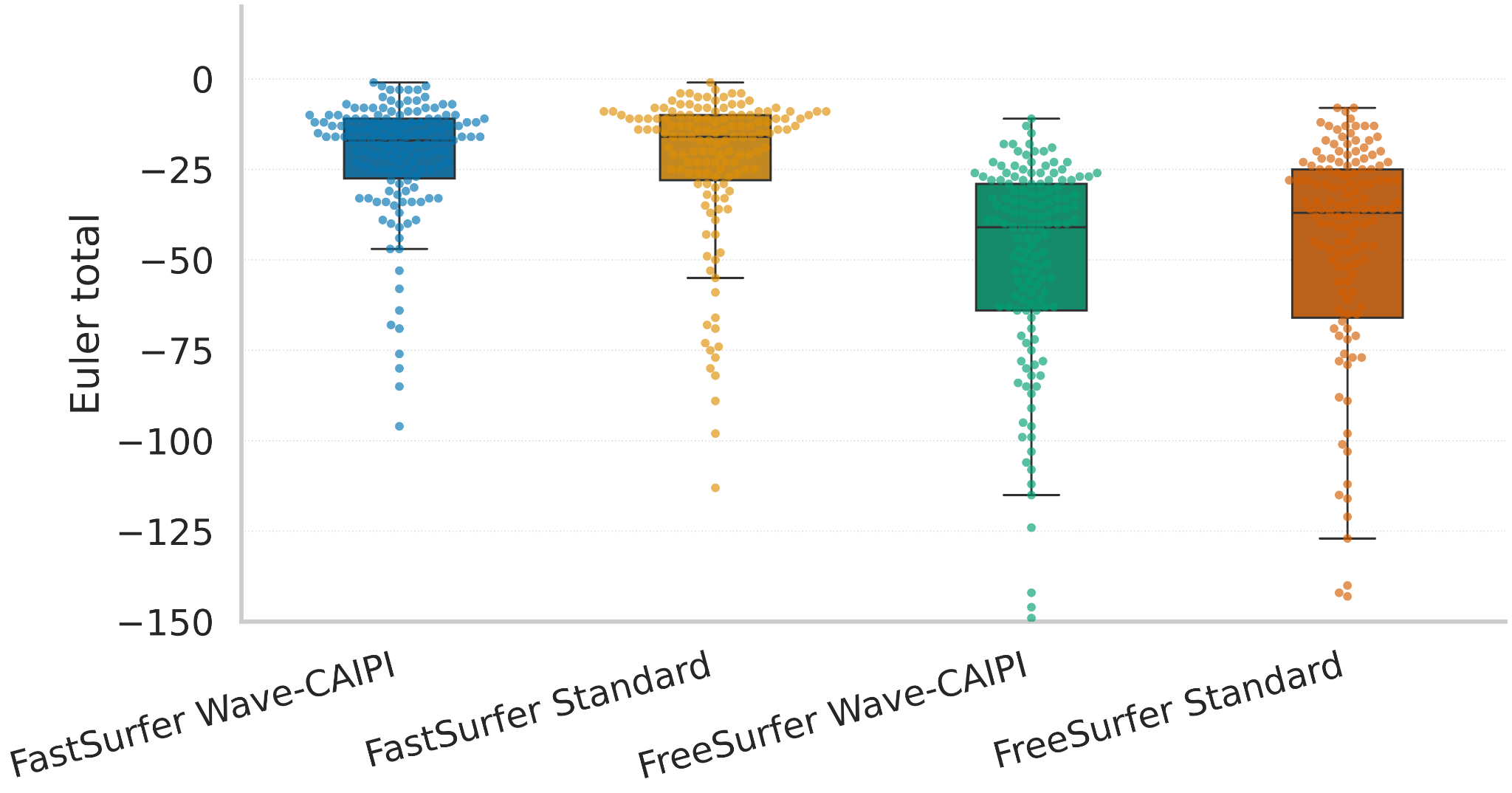


**Supplementary Figure 2.** Distribution of the Euler number produced by FreeSurfer and FastSurfer. Mann–Whitney U tests revealed no significant group differences between the Wave-CAIPI and standard acquisition protocol (FastSurfer: U = 10, 712.5, *p* = 0.90; FreeSurfer: U = 9 786.0, *p* = 0.16). Effect sizes were trivial (Cohen’s *d* = 0.13 and 0.09, respectively), indicating practical equivalence of the acquisition protocols for this metric.

| **Model** | **brainageR** | **DeepBrainNet** | **PyBrainAge** | **ENIGMA** | **pyment** | **MCCQR-MLP** |
| --- | --- | --- | --- | --- | --- | --- |
|  | **Standard MPRAGE** | | | | | |
| **MAE** | 5.98 [4.95, 7.02] | 6.47 [5.52, 7.44] | 7.46 [6.22, 8.73] | 9.06 [7.71, 10.44] | 4.66 [3.90, 5.46] | 6.89 [5.78, 8.04] |
| **r** | 0.72 [0.55, 0.83] | 0.68 [0.55, 0.79] | 0.57 [0.39, 0.70] | 0.41 [0.21, 0.58] | 0.81 [0.71, 0.87] | 0.76 [0.63, 0.85] |
| **R²** | 0.25 [-0.19, 0.52] | 0.22 [-0.17, 0.46] | -0.13 [-0.72, 0.23] | -0.56 [-1.29, -0.10] | 0.56 [0.33, 0.71] | 0.05 [-0.53, 0.41] |
|  | **Wave-CAIPI MPRAGE** | | | | | |
| **MAE** | 5.70 [4.69, 6.73] | 6.99 [5.92, 8.06] | 7.94 [6.62, 9.29] | 8.92 [7.52, 10.37] | 4.74 [4.00, 5.51] | 6.15 [5.16, 7.17] |
| **r** | 0.71 [0.55, 0.82] | 0.64 [0.49, 0.75] | 0.57 [0.40, 0.70] | 0.39 [0.19, 0.55] | 0.80 [0.71, 0.87] | 0.75 [0.62, 0.84] |
| **R²** | 0.31 [-0.13, 0.57] | 0.06 [-0.42, 0.36] | -0.28 [-0.96, 0.15] | -0.56 [-1.36, -0.06] | 0.56 [0.33, 0.71] | 0.24 [-0.21, 0.51] |

**Supplementary Table 3.** Performance metrics of the evaluated brain-age prediction models for standard MPRAGE and Wave-CAIPI MPRAGE acquisitions in the cross-sectional dataset on the NDD group.

**
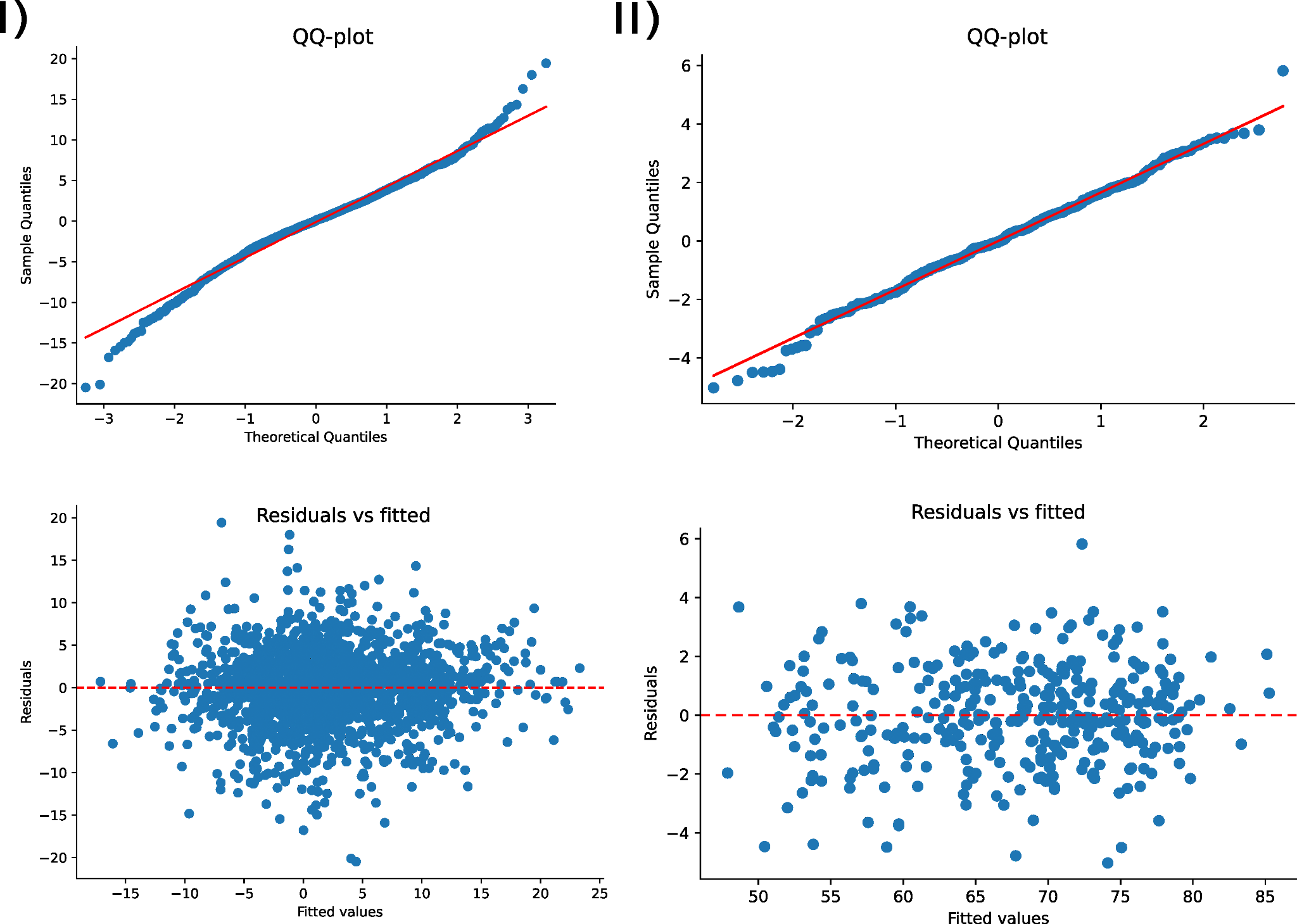
**

**Supplementary Figure 3.** Residual diagnostics for the mixed-effects models. I) RLMM fitted to the cross-sectional cohort (N = 147). The Q-Q plot (top) shows deviations from normality, consistent with the Shapiro–Wilk test (W = 0.983, *p* < 0.001). However, residuals showed no evidence of heteroscedasticity (Breusch–Pagan p = 0.13), and model robustness mitigates sensitivity to these deviations. II) LMM fitted to the short-interval longitudinal cohort (N = 15). Residuals were approximately normal (Shapiro–Wilk W = 0.997, p = 0.97), although the Breusch–Pagan test indicated some heteroscedasticity (p = 0.03). This was addressed by applying cluster-robust covariance estimation, adjusting standard errors to account for within-subject variance heterogeneity.

| **RLMM** | **Residual Min** | **Residual  1st Qu.** | **Residual Median** | **Residual Mean** | **Residual 3rd Qu.** | **Residual Max** |
| --- | --- | --- | --- | --- | --- | --- |
|  | 0.28 | 0.63 | 0.81 | 0.77 | 0.92 | 1.00 |

**Supplementary Table 4.** Summary of robustness weights for the residuals in the RLMM. The distribution of the down-weighted residuals is summarized by their minimum, quartiles, median, mean, and maximum values, reflecting the influence-limiting mechanism of the robust estimator.

| **Term** | **Estimate** | **SE** | **t value** | **p value** |
| --- | --- | --- | --- | --- |
| **Model brainageR** | 0.78 | 0.50 | 1.58 | 0.11 |
| **Model DeepBrainNet** | 2.23 | 0.50 | 4.48 | **< 0.001** |
| **Model PyBrainAge** | 2.83 | 0.50 | 5.70 | **< 0.001** |
| **Model ENIGMA** | 2.12 | 0.50 | 4.27 | **< 0.001** |
| **Model pyment** | 0.70 | 0.50 | 1.41 | 0.16 |
| **Model MCCQR-MLP** | 2.18 | 0.50 | 4.40 | **< 0.001** |
| **acq (Wave-CAIPI)** | -0.32 | 0.10 | -3.09 | **< 0.001** |
| **Disease Status (NDD)** | -3.46 | 0.50 | -6.97 | **< 0.001** |
| **Age** | -0.21 | 0.05 | -4.09 | **< 0.001** |
| **Sex[M]** | 0.03 | 0.55 | 0.05 | 0.96 |
| **ICV** | -3.18 | 3.35 | -0.95 | 0.34 |
| **Model1:acq (Wave-CAIPI)** | -1.24 | 0.23 | -5.40 | **< 0.001** |
| **Model2:acq (Wave-CAIPI)** | -0.03 | 0.23 | -0.13 | 0.90 |
| **Model3:acq (Wave-CAIPI)** | 0.34 | 0.23 | 1.48 | 0.14 |
| **Model4:acq (Wave-CAIPI)** | 1.09 | 0.23 | 4.76 | **< 0.001** |
| **Model5:acq (Wave-CAIPI)** | -0.21 | 0.23 | -0.90 | 0.37 |
| **Model1:Disease status (NDD)** | 0.63 | 0.26 | 2.45 | **0.01** |
| **Model2:Disease status (NDD)** | 0.15 | 0.26 | 0.57 | 0.57 |
| **Model3:Disease status (NDD)** | -0.43 | 0.26 | -1.67 | 0.10 |
| **Model4:Disease status (NDD)** | -0.79 | 0.26 | -3.03 | **< 0.001** |
| **Model5:Disease status (NDD)** | 0.89 | 0.26 | 3.42 | **< 0.001** |
| **acq (Wave-CAIPI):Disease status (NDD)** | -0.24 | 0.10 | -2.28 | **0.02** |
| **Model1:Age** | 0.12 | 0.03 | 4.47 | **< 0.001** |
| **Model2:Age** | 0.00 | 0.03 | 0.01 | 0.99 |
| **Model3:Age** | -0.04 | 0.03 | -1.57 | 0.12 |
| **Model4:Age** | -0.24 | 0.03 | -8.98 | **< 0.001** |
| **Model5:Age** | 0.06 | 0.03 | 2.36 | **0.02** |
| **Model1:Sex[M]** | -0.81 | 0.29 | -2.78 | **0.01** |
| **Model2:Sex[M]** | -0.16 | 0.29 | -0.55 | 0.58 |
| **Model3:Sex[M]** | 0.27 | 0.29 | 0.93 | 0.35 |
| **Model4:Sex[M]** | 0.38 | 0.29 | 1.30 | 0.19 |
| **Model5:Sex[M]** | -0.29 | 0.29 | -1.00 | 0.32 |
| **Model1:ICV** | 3.44 | 1.76 | 1.95 | 0.05 |
| **Model2:ICV** | -0.01 | 1.76 | -0.01 | 0.99 |
| **Model3:ICV** | -1.14 | 1.76 | -0.65 | 0.52 |
| **Model4:ICV** | -3.97 | 1.76 | -2.26 | **0.02** |
| **Model5:ICV** | 0.31 | 1.76 | 0.18 | 0.86 |
| **Model1:acq (Wave-CAIPI):Disease status (NDD)** | -0.41 | 0.23 | -1.80 | 0.07 |
| **Model2:acq (Wave-CAIPI):Disease status (NDD)** | -0.44 | 0.23 | -1.90 | 0.06 |
| **Model3:acq (Wave-CAIPI):Disease status (NDD)** | -0.15 | 0.23 | -0.64 | 0.52 |
| **Model4:acq (Wave-CAIPI):Disease status (NDD)** | 0.71 | 0.23 | 3.11 | **< 0.001** |
| **Model5:acq (Wave-CAIPI):Disease status (NDD)** | -0.25 | 0.23 | -1.10 | 0.27 |

**Supplementary Table 5.** Fixed effects estimates from the RLMM. The table reports the estimate, standard error, t-value, and corresponding p-value for each fixed effect term. Statistically significant effects (p < 0.05) are highlighted, with p-values < 0.001 reported as such. Interaction terms assess differential effects across acquisition protocols, SMC status, age, sex, and ICV across models.

| **Model** | **acq = Wave-CAIPI Estimate (SD)** | **Wave-CAIPI *p*** | **Standard MPRAGE Estimate (SD)** | **Standard MPRAGE *p*** |
| --- | --- | --- | --- | --- |
| **brainageR** | 6.95 (1.25) | **< 0.001** | 4.35 (1.21) | **< 0.001** |
| **DeepBrainNet** | 7.95 (1.25) | **< 0.001** | 5.28 (1.21) | **< 0.001** |

**Supplementary Table 6.** Simple contrasts for the effect of the clinical group on brain-age gap within each acquisition type across the models with a significant interaction term in the RLMM results. The table reports the estimated differences and corresponding p-values for the contrasts between SMC and NDD groups within the Wave-CAIPI MPRAGE and Standard MPRAGE acquisition types. Significant effects are highlighted in all four models, demonstrating a consistent influence of clinical group.

| **Model** | **Protocol** | **Cohen’s *d* [95 % CI]** | **AUC [95 % CI]** |
| --- | --- | --- | --- |
| **brainageR** | Standard | 0.53 [0.20, 0.85] | 0.66 [0.57, 0.74] |
|  | Wave-CAIPI | 0.77 [0.44, 1.11] | 0.75 [0.66, 0.82] |
| **DeepBrainNet** | Standard | 0.53 [0.20, 0.86] | 0.65 [0.56, 0.73] |
|  | Wave-CAIPI | 0.81 [0.48, 1.15] | 0.73 [0.65, 0.81] |
| **PyBrainAge** | Standard | 0.55 [0.22, 0.88] | 0.65 [0.56, 0.74] |
|  | Wave-CAIPI | 0.65 [0.32, 0.98] | 0.69 [0.60, 0.70] |
| **ENIGMA** | Standard | 0.51 [0.18, 0.84] | 0.65 [0.56, 0.74] |
|  | Wave-CAIPI | 0.32 [–0.01, 0.64] | 0.59 [0.51, 0.68] |
| **pyment** | Standard | 0.52 [0.19, 0.85] | 0.66 [0.57, 0.74] |
|  | Wave-CAIPI | 0.76 [0.42, 1.09] | 0.73 [0.65, 0.81] |
| **MCCQR‑MLP** | Standard | 0.96 [0.62, 1.31] | 0.78 [0.70, 0.85] |
|  | Wave-CAIPI | 0.82 [0.48, 1.16] | 0.74 [0.66, 0.82] |

**Supplementary Table 7.** Effect‑size (Cohen’s d) and discriminative accuracy (AUC) for separating participants with SMC from those with NDD across six brain‑age pipelines, shown for the standard MPRAGE and Wave‑CAIPI MPRAGE acquisition protocols. Point estimates are accompanied by 95 % bootstrap confidence intervals in parentheses.


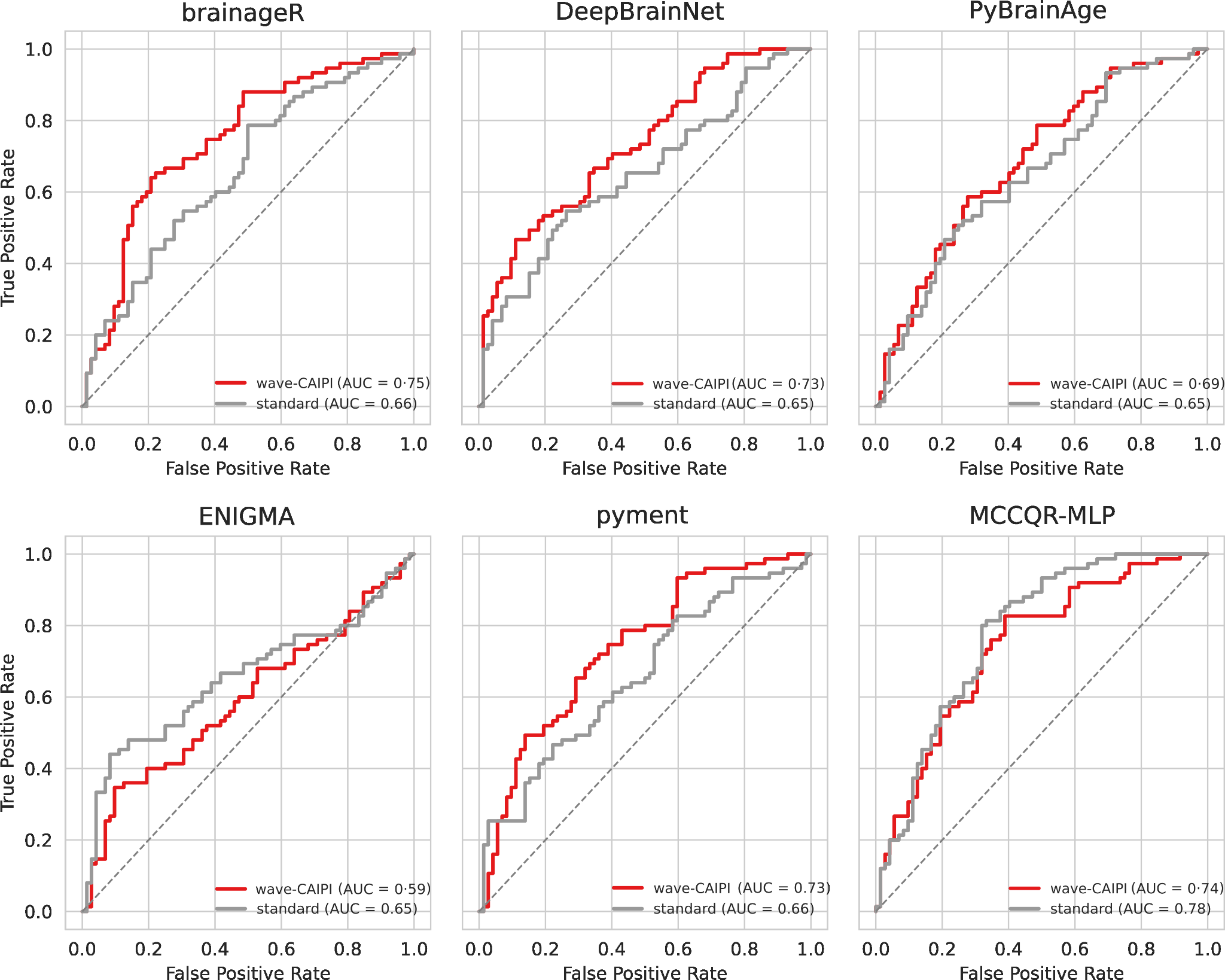


**Supplementary Figure 4.** ROC curves between SMC and NDD patients across the models evaluated. The performance of the Wave-CAIPI MPRAGE protocol (red) is compared to the standard MPRAGE protocol (gray). The Wave-CAIPI MPRAGE protocol demonstrates comparable or superior performance in brainageR, DeepBrainNet, PyBrainAge, and pyment models, while the standard MPRAGE protocol outperforms in ENIGMA and MCCQR-MLP. The dashed diagonal line indicates random classification performance.

| **Term** | **coef** | **SE** | ***t*** | ***p*** | **CI low** | **CI high** |
| --- | --- | --- | --- | --- | --- | --- |
| **brainageR** | 1.86 | 1.83 | 1.02 | 0.33 | -2.05 | 5.78 |
| **DeepBrainNet** | 0.97 | 1.82 | 0.53 | 0.60 | -2.93 | 4.86 |
| **ENIGMA** | -1.42 | 1.85 | -0.77 | 0.46 | -5.39 | 2.55 |
| **MCCQR-MLP** | 2.53 | 1.73 | 1.47 | 0.16 | -1.17 | 6.24 |
| **PyBrainAge** | -0.26 | 2.00 | -0.13 | 0.90 | -4.54 | 4.02 |
| **pyment** | 1.16 | 1.69 | 0.69 | 0.50 | -2.47 | 4.80 |
| **Disease status [NDD]** | -1.05 | 1.00 | -1.05 | 0.31 | -3.18 | 1.09 |
| **Sex[M]** | -0.41 | 1.58 | -0.26 | 0.80 | -3.80 | 2.97 |
| **session** | 0.90 | 0.54 | 1.68 | 0.12 | -0.25 | 2.06 |
| **Disease Status [NDD]: session** | -0.45 | 0.60 | -0.76 | 0.46 | -1.74 | 0.83 |
| **Age** | -0.09 | 0.04 | -2.20 | 0.05 | -0.17 | 0.00 |
| **ICV** | 0.95 | 2.48 | 0.38 | 0.71 | -4.36 | 6.26 |

**Supplementary Table 8.** Fixed effects from the LMM for the short-interval experiment. Coefficients are shown with their cluster-robust standard errors adjusted, t-values, p-values, and 95% confidence intervals.

|  |  | **Standard** | | | **Wave-CAIPI** | | |
| --- | --- | --- | --- | --- | --- | --- | --- |
| **Model** |  | **MAE** | **r** | **R²** | **MAE** | **r** | **R²** |
| **brainageR** | **SMC** | 2.83 [1.75, 4.09] | 0.88 [0.79, 0.97] | 0.68 [-0.46, 0.90] | 5.71 [3.87, 7.43] | 0.75 [0.57, 0.95] | -0.09 [-2.69, 0.29] |
|  | **NDD** | 4.70 [2.46, 7.24] | 0.60 [0.24, 0.90] | -0.11 [-1.40, 0.64] | 5.15 [3.38, 7.06] | 0.69 [0.48, 0.86] | 0.05 [-0.86, 0.47] |
| **DeepBrainNet** | **SMC** | 5.44 [3.99, 6.76] | 0.65 [0.40, 0.88] | 0.08 [-2.68, 0.41] | 3.78 [2.38, 5.13] | 0.71 [0.52, 0.92] | 0.48 [-0.44, 0.66] |
|  | **NDD** | 5.82 [4.16, 7.44] | 0.72 [0.53, 0.88] | -0.05 [-1.03, 0.35] | 6.10 [4.29, 7.97] | 0.63 [0.42, 0.79] | -0.20 [-1.38, 0.34] |
| **PyBrainAge** | **SMC** | 7.30 [3.93, 11.19] | 0.06 [-0.40, 0.86] | -1.45 [-3.60, -0.74] | 7.30 [3.91, 10.90] | 0.16 [-0.23, 0.78] | -1.37 [-3.89, -0.60] |
|  | **NDD** | 5.77 [3.76, 7.84] | 0.46 [0.14, 0.69] | -0.19 [-1.36, 0.36] | 6.11 [3.70, 8.78] | 0.49 [0.09, 0.78] | -0.55 [-2.55, 0.37] |
| **ENIGMA** | **SMC** | 8.39 [4.38, 13.03] | 0.00 [-0.63, 0.69] | -2.34 [-5.90, -1.04] | 8.21 [4.17, 12.70] | 0.04 [-0.40, 0.65] | -2.13 [-6.20, -0.88] |
|  | **NDD** | 9.28 [7.22, 11.32] | 0.25 [-0.13, 0.54] | -1.40 [-3.74, -0.39] | 10.00 [7.46, 12.38] | 0.20 [-0.16, 0.50] | -1.90 [-4.75, -0.68] |
| **pyment** | **SMC** | 2.65 [1.51, 3.87] | 0.85 [0.35, 0.96] | 0.71 [-0.46, 0.91] | 3.03 [1.97, 4.04] | 0.86 [0.60, 0.97] | 0.68 [-0.33, 0.85] |
|  | **NDD** | 5.66 [4.32, 6.92] | 0.52 [0.19, 0.81] | 0.09 [-0.53, 0.35] | 5.26 [3.99, 6.57] | 0.57 [0.26, 0.79] | 0.20 [-0.45, 0.51] |
| **MCCQR-MLP** | **SMC** | 6.70 [5.03, 8.49] | 0.43 [0.05, 0.73] | -0.41 [-3.49, 0.24] | 5.54 [3.44, 7.58] | 0.71 [0.44, 0.91] | -0.12 [-3.09, 0.41] |
|  | **NDD** | 5.37 [3.78, 7.12] | 0.84 [0.73, 0.94] | 0.04 [-1.10, 0.55] | 3.96 [2.63, 5.34] | 0.80 [0.68, 0.90] | 0.46 [-0.13, 0.74] |

**Supplementary Table 9.** Performance metrics of the six brain-age prediction models applied to the short-interval longitudinal dataset, separately for Standard and Wave-CAIPI acquisition protocols. MAE, r, and R² values are reported with 95% confidence intervals calculated via bootstrapping (5000 repetitions). Pyment consistently yielded the best performance across both protocols, while ENIGMA and PyBrainAge showed lower accuracy and explained variance, particularly under the Wave-CAIPI protocol.

**
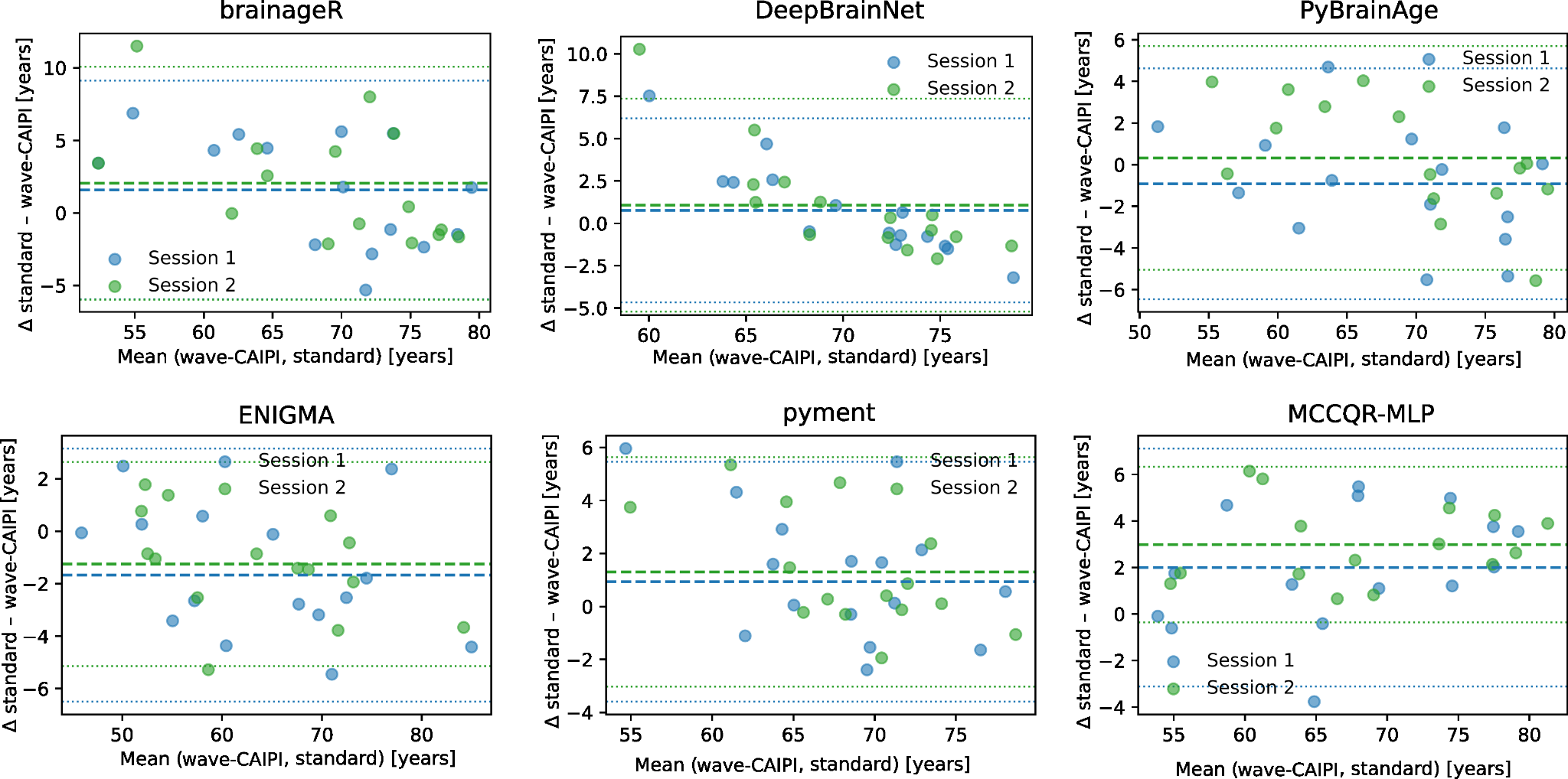
**

**Supplementary Figure 5.** Bland–Altman plots comparing Standard and Wave-CAIPI brain-age across sessions. The y-axis shows the difference in predicted age (Δ = Standard – Wave-CAIPI), and the x-axis shows the mean predicted age across protocols. Dashed lines indicate the mean difference and ±1.96 SD limits. Pyment and brainageR show the smallest protocol-related discrepancies, while ENIGMA and PyBrainAge exhibit greater variability and larger biases.

## **Supplementary materials – References**

Alexander, B., Loh, W. Y., Matthews, L. G., Murray, A. L., Adamson, C., Beare, R., et al. (2019). Desikan-Killiany-Tourville Atlas Compatible Version of M-CRIB Neonatal Parcellated Whole Brain Atlas: The M-CRIB 2.0. *Front. Neurosci.* 13. doi: 10.3389/fnins.2019.00034

Ernsting, J., Winter, N. R., Leenings, R., Sarink, K., Barkhau, C. B. C., Fisch, L., et al. (2023). From Group-Differences to Single-Subject Probability: Conformal Prediction-based Uncertainty Estimation for Brain-Age Modeling. doi: 10.48550/arXiv.2302.05304

Hahn, T., Ernsting, J., Winter, N. R., Holstein, V., Leenings, R., Beisemann, M., et al. (2022). An uncertainty-aware, shareable, and transparent neural network architecture for brain-age modeling. *Science advances* 8, eabg9471.

Henschel, L., Conjeti, S., Estrada, S., Diers, K., Fischl, B., and Reuter, M. (2020). FastSurfer - A fast and accurate deep learning based neuroimaging pipeline. *NeuroImage* 219, 117012. doi: 10.1016/j.neuroimage.2020.117012

IXI Dataset – Brain Development (n.d.). Available at: https://brain-development.org/ixi-dataset/ (Accessed September 19, 2025).

Johnson, W. E., Li, C., and Rabinovic, A. (2007). Adjusting batch effects in microarray expression data using empirical Bayes methods. *Biostatistics* 8, 118–127. doi: 10.1093/biostatistics/kxj037

Maia Polo, F., and Vicente, R. (2023). Effective sample size, dimensionality, and generalization in covariate shift adaptation. *Neural Comput & Applic* 35, 18187–18199. doi: 10.1007/s00521-021-06615-1

Marcus, D. S., Wang, T. H., Parker, J., Csernansky, J. G., Morris, J. C., and Buckner, R. L. (2007). Open Access Series of Imaging Studies (OASIS): Cross-sectional MRI Data in Young, Middle Aged, Nondemented, and Demented Older Adults. *J Cogn Neurosci* 19, 1498–1507. doi: 10.1162/jocn.2007.19.9.1498

Nárai, Á., Hermann, P., Auer, T., Kemenczky, P., Szalma, J., Homolya, I., et al. (2022). Movement-related artefacts (MR-ART) dataset of matched motion-corrupted and clean structural MRI brain scans. *Sci Data* 9, 630. doi: 10.1038/s41597-022-01694-8

Navarro-González, R., García-Azorín, D., Guerrero-Peral, Á. L., Planchuelo-Gómez, Á., Aja-Fernández, S., and de Luis-García, R. (2023). Increased MRI-based Brain Age in chronic migraine patients. *J Headache Pain* 24, 133. doi: 10.1186/s10194-023-01670-6

Nooner, K. B., Colcombe, S., Tobe, R., Mennes, M., Benedict, M., Moreno, A., et al. (2012). The NKI-Rockland Sample: A Model for Accelerating the Pace of Discovery Science in Psychiatry. *Front. Neurosci.* 6. doi: 10.3389/fnins.2012.00152

Nugent, A. C., Thomas, A. G., Mahoney, M., Gibbons, A., Smith, J. T., Charles, A. J., et al. (2022). The NIMH intramural healthy volunteer dataset: A comprehensive MEG, MRI, and behavioral resource. *Sci Data* 9, 518. doi: 10.1038/s41597-022-01623-9

Park, D. C., Hennessee, J. P., Smith, E. T., Chan, M. Y., Chen, X., Dakanali, M., et al. (2025). The Dallas Lifespan Brain Study: A Comprehensive Adult Lifespan Data Set of Brain and Cognitive Aging. *Sci Data* 12, 846. doi: 10.1038/s41597-025-04847-7

Shafto, M. A., Tyler, L. K., Dixon, M., Taylor, J. R., Rowe, J. B., Cusack, R., et al. (2014). The Cambridge Centre for Ageing and Neuroscience (Cam-CAN) study protocol: a cross-sectional, lifespan, multidisciplinary examination of healthy cognitive ageing. *BMC Neurol* 14, 204. doi: 10.1186/s12883-014-0204-1

Spreng, R. N., Setton, R., Alter, U., Cassidy, B. N., Darboh, B., DuPre, E., et al. (2022). Neurocognitive aging data release with behavioral, structural and multi-echo functional MRI measures. *Sci Data* 9, 119. doi: 10.1038/s41597-022-01231-7

Taylor, J. R., Williams, N., Cusack, R., Auer, T., Shafto, M. A., Dixon, M., et al. (2017). The Cambridge Centre for Ageing and Neuroscience (Cam-CAN) data repository: Structural and functional MRI, MEG, and cognitive data from a cross-sectional adult lifespan sample. *NeuroImage* 144, 262–269. doi: 10.1016/j.neuroimage.2015.09.018

Tisdall, L., Mugume, S., Kellen, D., and Mata, R. (2024). Lifespan trajectories of risk preference, impulsivity, and self-control: A dataset containing self-report, informant-report, behavioral, hormone and functional neuroimaging measures from a cross-sectional human sample. *Data in Brief* 52, 109968. doi: 10.1016/j.dib.2023.109968

Wei, D., Zhuang, K., Ai, L., Chen, Q., Yang, W., Liu, W., et al. (2018). Structural and functional brain scans from the cross-sectional Southwest University adult lifespan dataset. *Sci Data* 5, 180134. doi: 10.1038/sdata.2018.134

Zuo, X.-N., Anderson, J. S., Bellec, P., Birn, R. M., Biswal, B. B., Blautzik, J., et al. (2014). An open science resource for establishing reliability and reproducibility in functional connectomics. *Sci Data* 1, 140049. doi: 10.1038/sdata.2014.49
